# Protection of melatonin treatment and combination with traditional antibiotics against septic myocardial injury

**DOI:** 10.1186/s11658-022-00415-8

**Published:** 2023-04-26

**Authors:** Wencheng Di, Zhenxiao Jin, Wangrui Lei, Qiong Liu, Wenwen Yang, Shaofei Zhang, Chenxi Lu, Xiaoling Xu, Yang Yang, Huadong Zhao

**Affiliations:** 1 Department of Cardiovascular Medicine, National Clinical Research Center for Infectious Disease, Shenzhen Third People’s Hospital, 29 Bulan Road, Shenzhen, Guangdong Province, China; 2 Department of Cardiovascular Surgery, Xijing Hospital, The Airforce Military Medical University, 127 Changle West Road, Xi’an, China; 3 Key Laboratory of Resource Biology and Biotechnology in Western China, Ministry of Education, 229 Taibai North Road, Xi’an, China; 4 Faculty of Life Sciences and Medicine, Northwest University, 229 Taibai North Road, Xi’an, China; 5 Department of General Surgery, Tangdu Hospital, The Airforce Military Medical University, 1 Xinsi Road, Xi’an, China

**Keywords:** Melatonin, Septic myocardial injury, RNA-seq, Pretreatment, Posttreatment, Antibiotics

## Abstract

**Background:**

Heart failure is a common complication of sepsis with a high mortality rate. It has been reported that melatonin can attenuate septic injury due to various properties. On the basis of previous reports, this study will further explore the effects and mechanisms of melatonin pretreatment, posttreatment, and combination with antibiotics in the treatment of sepsis and septic myocardial injury.

**Methods and results:**

Our results showed that melatonin pretreatment showed an obvious protective effect on sepsis and septic myocardial injury, which was related to the attenuation of inflammation and oxidative stress, the improvement of mitochondrial function, the regulation of endoplasmic reticulum stress (ERS), and the activation of the AMPK signaling pathway. In particular, AMPK serves as a key effector for melatonin-initiated myocardial benefits. In addition, melatonin posttreatment also had a certain degree of protection, while its effect was not as remarkable as that of pretreatment. The combination of melatonin and classical antibiotics had a slight but limited effect. RNA-seq detection clarified the cardioprotective mechanism of melatonin.

**Conclusion:**

Altogether, this study provides a theoretical basis for the application strategy and combination of melatonin in septic myocardial injury.

**Supplementary Information:**

The online version contains supplementary material available at 10.1186/s11658-022-00415-8.

## Introduction

Sepsis can further develop into multiple organ failure [1], and the heart is one of the most vulnerable organs [2]. sepsis is characterized by profound and sustained inflammatory response, but previous clinical trials of anti-inflammatory therapies to counteract excessive inflammation in patients with sepsis are unsatisfying. There remains a need to provide therapeutic targets or strategies in preclinical settings. Melatonin is an endogenous indoleamine compound that regulates the circadian rhythm [3] and plays an active role in various cardiovascular diseases due to the ability of antioxidation, anti-inflammation, and anti-apoptosis [4]. There is also evidence that melatonin can effectively inhibit septic myocardial injury via improving mitochondrial function [5–8]. Unsatisfactorily, few studies have been conducted to compare the protective effects of melatonin before and after administration, and to evaluatethe synergistic effect of melatonin combination with azithromycin or vancomycin on sepsis and septic myocardial injury.

AMP-activated protein kinase (AMPK) is a member of the serine/threonine kinase family, which monitors the AMP-to-ATP ratio at the cellular and whole-organism level [9]. Studies have confirmed that AMPK is involved in myocardial ischemia–reperfusion injury [10], myocardial hypertrophy [11], and myocardial fibrosis [12]. Notably, an important study confirmed that melatonin induces cardioprotection via the activation of the AMPK-dependent autophagy pathway in lipopolysaccharide (LPS)-induced mice [13]. However, the exact mechanism remains unclear.

Given that heart failure is the primary cause of death in sepsis [14], our study aims to explore: (1) melatonin alone or in combination with classic antibiotics for the treatment of sepsis and septic myocardial injury; (2) the specific roles and potential molecular mechanisms of AMPK pathway in melatonin against septic myocardial injury; (3) the comprehensive protective mechanisms of melatonin by RNA-seq detection.

## Materials and methods

### Animals

Male Balb/c mice aged 8–10 weeks were obtained from the animal center of Airforce Medical University (Xi’an, Shaanxi, China). All mice had free access to food and water and were bred at 26 ℃ in a 12 h light /12 h dark cycle. All animal experiment protocols were conducted in accordance with the guidelines of the Animal Care and Use Committees at Northwest University (approval no. 2019025, Xi’an, Shaanxi, China) and were in compliance with the Guidelines for the Care and Use of Laboratory Animals (NIH publication no. 85–23, revised 2011).

### Cecal ligation and puncture (CLP) model

CLP model was performed according to our previous study [15]. Briefly, the cecum was tightly ligated at 1/3 site from its end using 4–0 nylon suture, and double punctures of the cecal wall were performed with a 25 G needle (Additional file 1: Fig. S1a and Table S1). For a survival experiment, an aggravated CLP model was constructed. The cecum was tightly ligated at 2/3 site from its end (Additional file 1: Fig. S1b). Sepsis score was conducted by two investigators after the induction of sepsis for 8 h by murine sepsis score (MSS). Anal temperature was determined at post-CLP 8 h by using an animal thermometer (Calvin Biotechnology Co., Ltd., Nanjing, Jiangsu, China).

### Experimental design

#### Pretreatment experiment

**Fig. 1:**
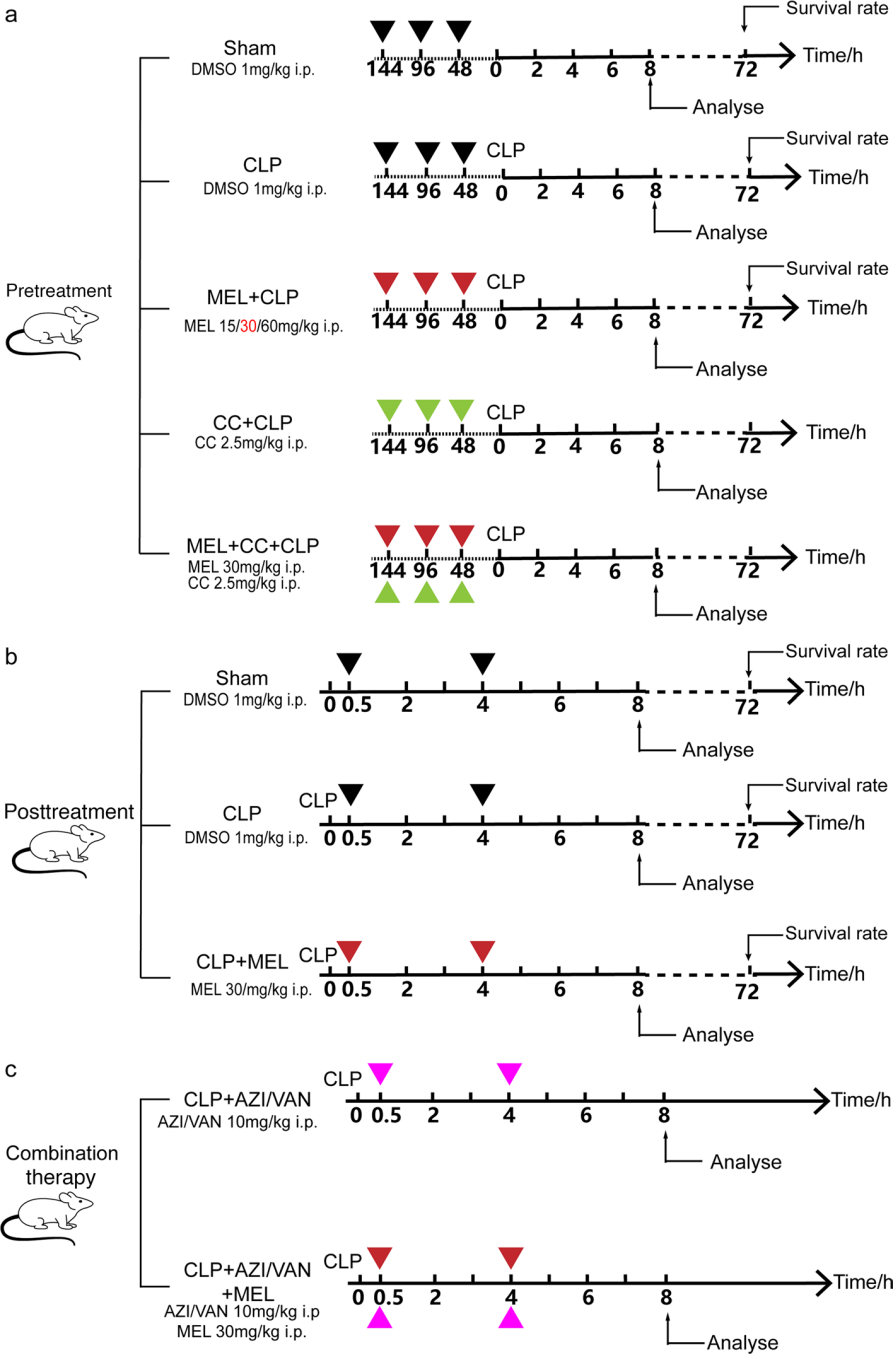
Schematic illustration of the present study. **a** Pretreatment experiment. Melatonin or Compound C was dissolved in DMSO and given to mice every 2 days for 6 days before CLP injury. Mice were randomly allocated into the following groups: (1) mice injected with 1 ml/kg DMSO and subjected to sham surgery; (2) mice injected with 1 ml/kg DMSO and then subjected to CLP surgery; (3) mice treated with melatonin and then subjected to CLP surgery. In the third group, different concentrations of melatonin (15 mg/kg, 30 mg/kg, or 60 mg/kg) were administered to mice to observe the effect of MEL on survival rate within 72 h, among which 30 mg/kg was selected for further analysis, including cardiac function, biochemistry, and molecular parameters. For Compound C (CC) experiment, two groups were added: (4) mice injected with 30 mg/kg melatonin and 2.5 mg/kg CC and then subjected to CLP surgery; (5) mice injected with 2.5 mg/kg CC and then subjected to CLP surgery. **b** Posttreatment experiment. Melatonin was dissolved in DMSO and given to mice at 30 min and 4 h post CLP. Mice were randomly allocated into the following groups: (1) mice subjected to sham surgery and then injected with 1 ml/kg DMSO; (2) mice subjected to CLP surgery and then injected with 1 mg/kg DMSO; (3) mice subjected to CLP surgery and treated with 30 mg/kg melatonin. **c** Combination therapy. Melatonin, azithromycin, or vancomycin were dissolved in DMSO and given to mice at 30 min and 4 h post CLP. Mice were randomly allocated into the following groups: (1) mice subjected to CLP surgery and then treated with 10 mg/kg azithromycin (or vancomycin); (2) mice subjected to CLP surgery and then treated with 10 mg/kg (or vancomycin) and 30 mg/kg melatonin

Melatonin or Compound C was dissolved in dimethyl sulfoxide (DMSO) and given to mice every 2 days for 6 days before CLP injury. Mice were allocated into the following groups: (a) mice injected with 1 ml/kg DMSO and subjected to sham surgery; (b) mice injected with 1 ml/kg DMSO and then subjected to CLP surgery; (c) mice treated with melatonin and then subjected to CLP surgery. In the third group, different concentrations of melatonin (15 mg/kg, 30 mg/kg, or 60 mg/kg) were administered to mice to observe the effect of melatonin on the survival rate within 72 h, among which 30 mg/kg was selected for further analysis, including cardiac function, biochemistry, and molecular parameters (Fig. 1a). For Compound C (CC) experiment, two groups were added: (d) mice injected with 30 mg/kg melatonin and 2.5 mg/kg CC and then subjected to CLP surgery; (e) mice injected with 2.5 mg/kg CC and then subjected to CLP surgery (Fig. 1a).

#### Posttreatment experiment

Melatonin was dissolved in DMSO and given to mice at 30 min and 4 h post CLP. Mice were allocated into the following groups: (a) mice subjected to sham surgery and then injected with 1 ml/kg DMSO; (b) mice subjected to CLP surgery and then injected with 1 mg/kg DMSO; (c) mice subjected to CLP surgery and administered 30 mg/kg melatonin(Fig. 1b).

#### Combination experiment

Melatonin, azithromycin, or vancomycin were dissolved in DMSO and given to mice at 30 min and 4 h post CLP. Mice were randomly allocated into the following groups: (a) mice subjected to CLP surgery and then treated with 10 mg/kg azithromycin (or vancomycin); (b) mice subjected to CLP surgery and then treated with 10 mg/kg azithromycin (or vancomycin) and 30 mg/kg melatonin.  (Fig. 1c).

### Statistical analysis

Data were analyzed by using GraphPad Prism 9.0.0 (GraphPad Software Inc., San Diego, CA, USA). All values are presented as mean ± standard deviation (SD). To test for statistically significant differences, *t*-test was used for any two groups, and one-way ANOVA followed by Tukey’s test was used for more than two groups. Asterisks are used to represent significance, and the level of significance is represented as follows: *P < 0.05, **P < 0.01, ***P < 0.001, *P* < 0.0001.

Additional information on materials and methods can be found in Additional file 1.

## Results

### Protective effect of melatonin pretreatment against sepsis and septic myocardial injury in mice

**Fig. 2:**
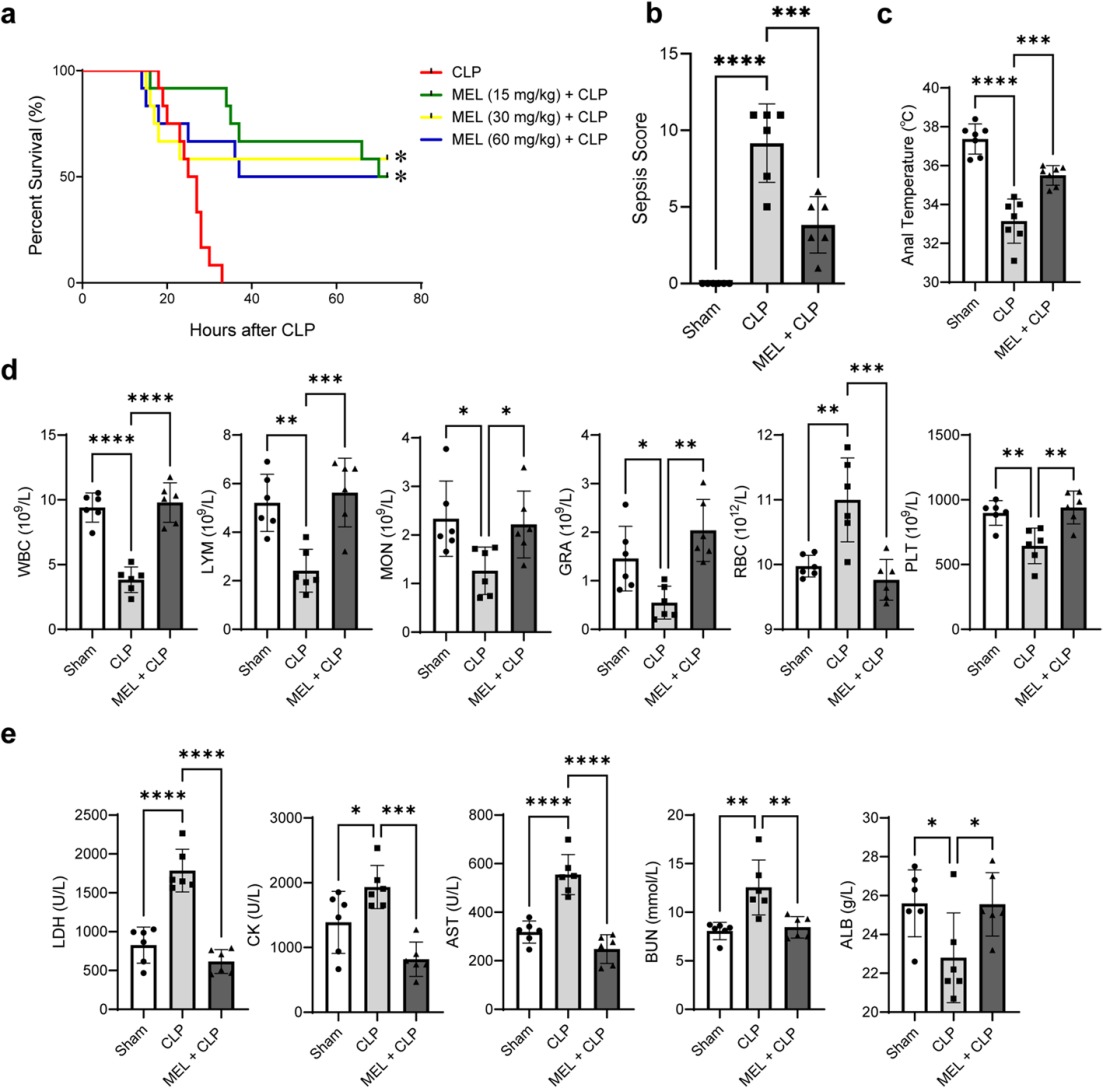
Protective effect of melatonin pretreatment in septic mice. **a** Kaplan–Meier survival curves. Twelve animals for each group were used for comparison. Mortality was observed within 72 h. **b** Sepsis scores (*n* = 6 for each group). **c** Anal temperature (*n* = 7 for each group). **d** Blood routine parameters (*n* = 6 for each group). **e** Blood biochemical parameters (*n* = 6 for each group). ^*^P < 0.05, **P < 0.01, ***P < 0.001, ****P < 0.0001 versus sham or versus CLP; *ns,* nonsignificant. Statistical analysis of data was performed using ANOVA

Here, we first observed the efficacy of melatonin pretreatment in CLP mice. Mice were given different concentrations of melatonin (15, 30, and 60 mg/kg) to observe the survival rate within 72 h of CLP. All mice in the CLP group died within 36 h, while the survival rates of the MEL + CLP groups were improved, among which 30 mg/kg melatonin pretreatment exhibited the best effect (Fig. 2a), and thus this concentration was selected for further studies. The decreased sepsis score while the increased anal temperature in the MEL + CLP group further demonstrated the survival advantage of melatonin (Fig. 2b, c). In addition, to explore the influence of melatonin on systemic inflammation and multiple organ failure, assessment of blood routine and blood biochemical parameters was performed. Melatonin could increase the numbers of circulating inflammatory cells [white blood cells (WBC), lymphocytes (LYM), monocyte (MON), and granulocytes (GRA)] and platelets (PLT) (Fig. 2d) as well as decrease the levels of red blood cells (RBC) in CLP mice (Fig. 2d), indicating that melatonin enhances the host’s immunity capacity. These changes of blood routine parameters were accompanied by reductions in the biochemical markers of organ injury, especially cardiac injury (Fig. 2e), such as lactic dehydrogenase (LDH), creatine kinase (CK), and aspartate aminotransferase (AST).

**Fig. 3:**
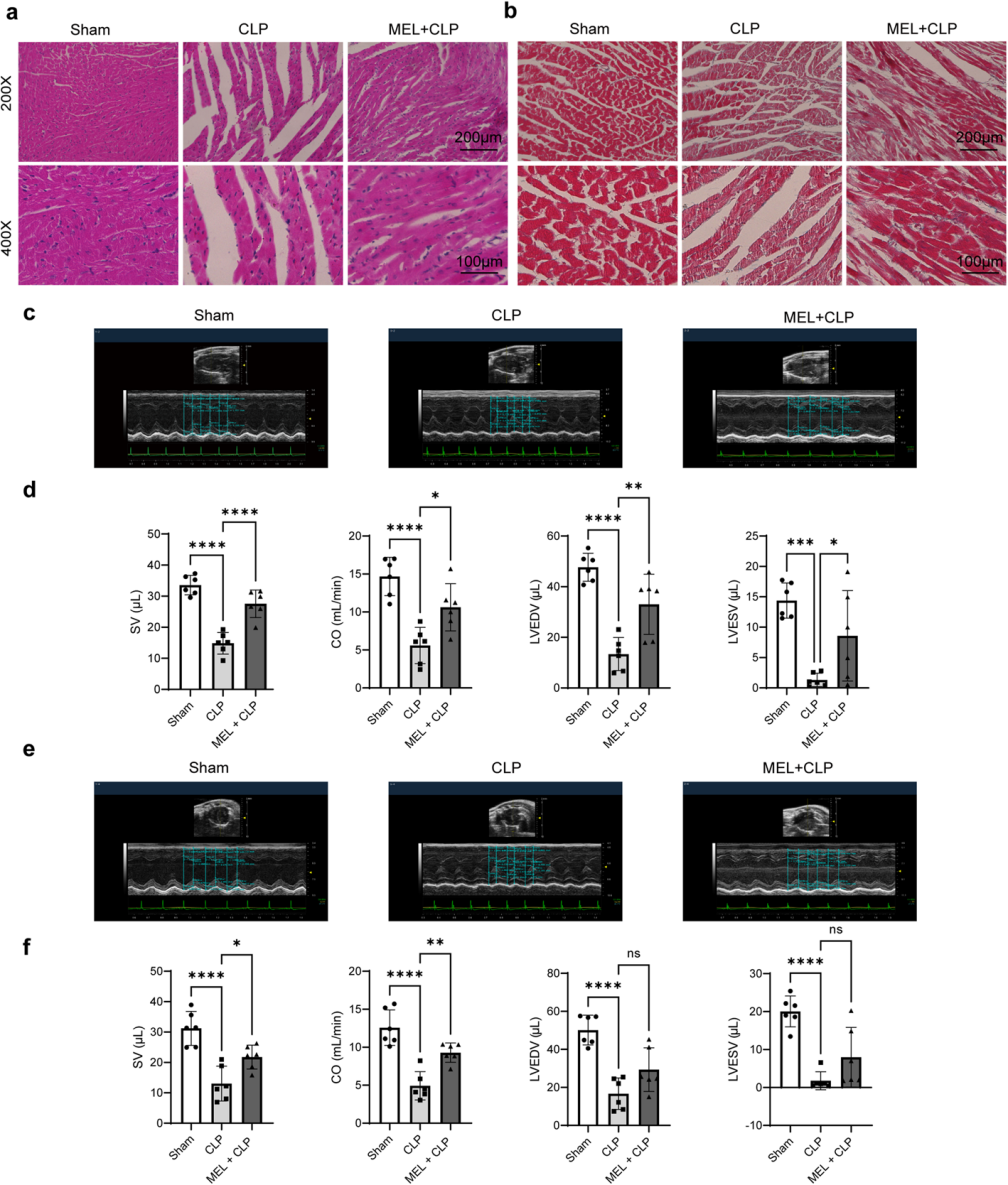
Protective effect of melatonin pretreatment against septic myocardial injury in mice. **a** Representative images of H&E staining of mouse heart tissues. **b** Representative images of Masson’s trichrome staining of mouse heart tissues. **c** Representative echocardiography images of the long axis. **d** SV, CO, LVEDV, and LVESV statistical graphs of the long axis. **e** Representative echocardiography images of the short axis. **f** SV, CO, LVEDV, and LVESV statistical graphs of the short axis. *P < 0.05, **P < 0.01, ***P < 0.001, ****P < 0.0001 versus sham or versus CLP; *ns,* non-significant. *n* = 6 for each group. Statistical analysis of data was performed using ANOVA

Then, the myocardial structure and the cardiac function were examined. There was more severe myocardial tissue damage in CLP-injured mice, manifested as disrupted myocardial fibers, aggravated interstitial edema, and destruction of cellular integrity, the effects of which were reversed by melatonin (Fig. 3a). Masson’s staining showed that cardiac fibrosis was elevated in CLP mice but improved in MEL + CLP mice (Fig. 3b). In particular, as shown in Fig. 3c–f, the echocardiography revealed a significant reduction of stroke volume (SV), cardiac output (CO), left ventricular diastolic volume (LVEDV), and left ventricular systolic volume (LVESV) in CLP. As anticipated, melatonin increased these cardiac function parameters, showing its strong cardioprotective effects. Additional echocardiographic data are presented in Additional file 1: Fig. S2.

### Effect of melatonin pretreatment on inflammation, oxidative stress, endoplasmic reticulum stress (ERS), mitochondrial function, and the AMPK signaling pathway in septic mice

**Fig. 4:**
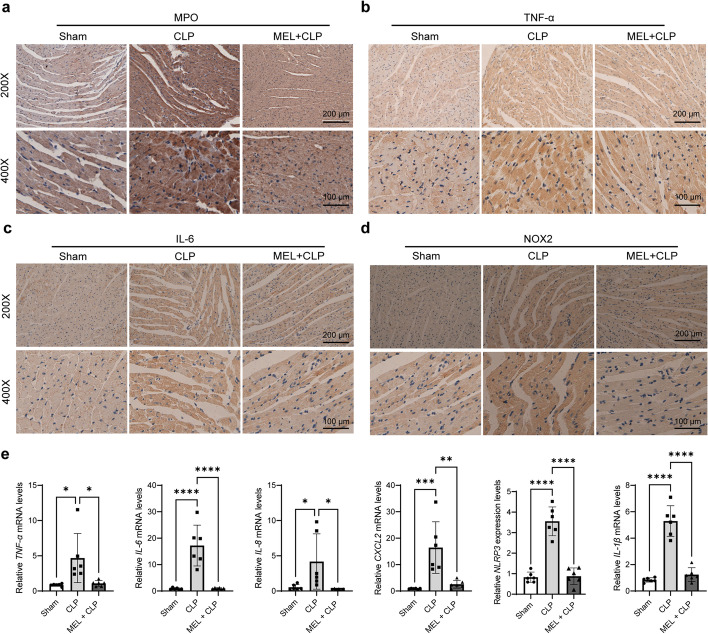
Effect of melatonin pretreatment on myocardial inflammation in septic mice. Representative photographs of immunohistochemical staining of MPO (**a**), TNF-α (**b**), IL-6 (**c**), and NOX2 (**d**) in mouse heart tissues. **e** qRT-PCR analysis of *TNF-α,* *IL-6*, *IL-8*, *CXCL2*, *NLRP3*, and *IL-1β*. *P < 0.05, **P < 0.01, ***P < 0.001, ****P < 0.0001 versus sham or versus CLP; *ns,* nonsignificant. *n* = 6 for each group. Statistical analysis of data was performed using ANOVA

Sepsis induces an amplified abnormal state, including inflammation, oxidative stress, and mitochondrial dysfunction [16]. Inflammation exerts a vital role in the cardiac dysfunction during sepsis. The levels of MPO, TNF-α, and IL-6 in myocardium were measured by immunohistochemistry (IHC) staining. MPO is a marker of neutrophil aggregation. The result showed that CLP injury led to increased MPO contents in the heart, but the extent was much less dramatic in the CLP + MEL group (Fig. 4a). Similarly, the contents of TNF-α, IL-6, and NOX2 displayed a similar trend (Fig. 4b–d). Melatonin pretreatment also decreased the mRNA expression of *TNF-α*, *IL-6*, *IL-8*, *CXCL*2, *NLRP3*, and *IL-1β* in CLP mice(Fig. 4e).

**Fig. 5:**
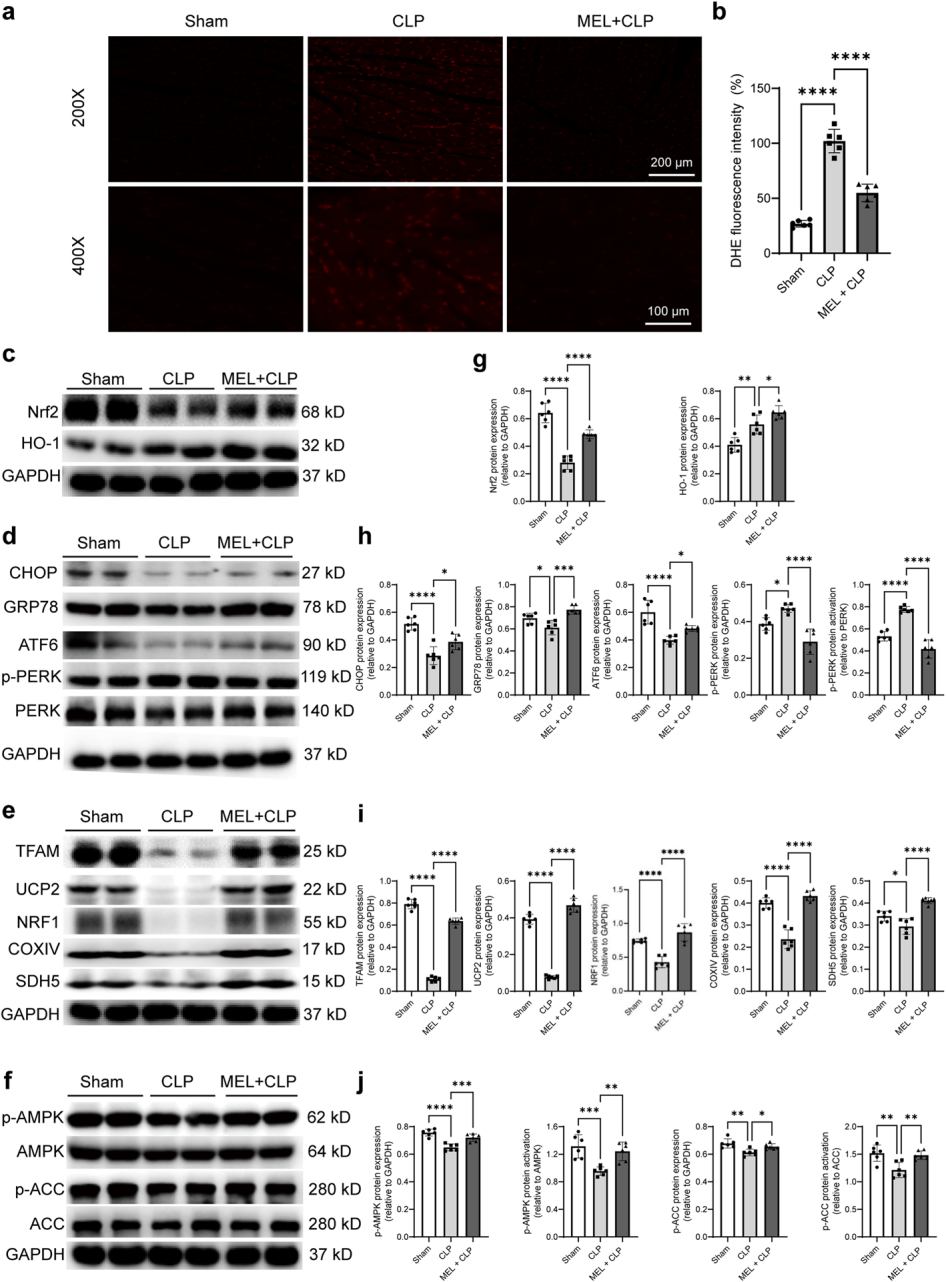
Effect of melatonin pretreatment on oxidative stress, endoplasmic reticulum stress, mitochondrial function, and the AMPK/ACC signaling pathway in septic mice. **a** Representative photographs of DHE staining. **b** Quantitative analysis of DHE staining. **c**–**f** Representative western blot protein bands of Nrf2, HO-1, CHOP, GRP78, ATF6, p-PERK, PERK, TFAM, UCP2, NRF1, COXIV, SDH5, p-AMPK, AMPK, p-ACC, and ACC. **g**–**j** Quantitative analysis of western blot by normalizing to GAPDH. ^*^P < 0.05, ^**^P < 0.01, ^***^P < 0.001, ^****^P < 0.0001 versus sham or versus CLP; *ns,* nonsignificant. *n* = 6 for each group. Statistical analysis of data was performed using ANOVA

As oxidative stress is correlated with organ failure in sepsis, cardiac reactive oxygen species (ROS) level and the known antioxidative stress markers Nrf2 and HO-1 in the heart tissues were examined. Increased red fluorescent sites were observed in the CLP group, while melatonin decreased the red fluorescent sites (Fig. 5a–b). Consistently, melatonin also up-regulated the protein level of Nrf2 and HO-1of CLP mice (Fig. 5c, g).

As prolonged ERS has been demonstrated to be a crucial contributor to sepsis, the levels of ERS-related markers were then assessed. The levels of CHOP, GRP78, and ATF6 were significantly reduced, while the levels of p-PERK and p-PERK/PERK were increased in the CLP group, the effects of which were reversed by melatonin (Fig. 5d, h).

Mitochondrial dysfunction has been also observed during sepsis. The protein levels of TFAM, UCP2, NRF1, COXIV, and SDH5 were all significantly reduced in the CLP group, while melatonin could upregulate those protein levels (Fig. 5e, i). In addition, the AMPK pathway, a classical cardioprotective pathway,  is closely correlated with mitochondrial biogenesis and function [17]. In line with improved mitochondrial function, CLP-induced reduction of p-AMPK, p-AMPK/AMPK, p-ACC, and p-ACC/ACC was restored by melatonin pretreatment (Fig. 5f, j).

### Effect of CC on the protective role of melatonin pretreatment in septic mice

**Fig. 6:**
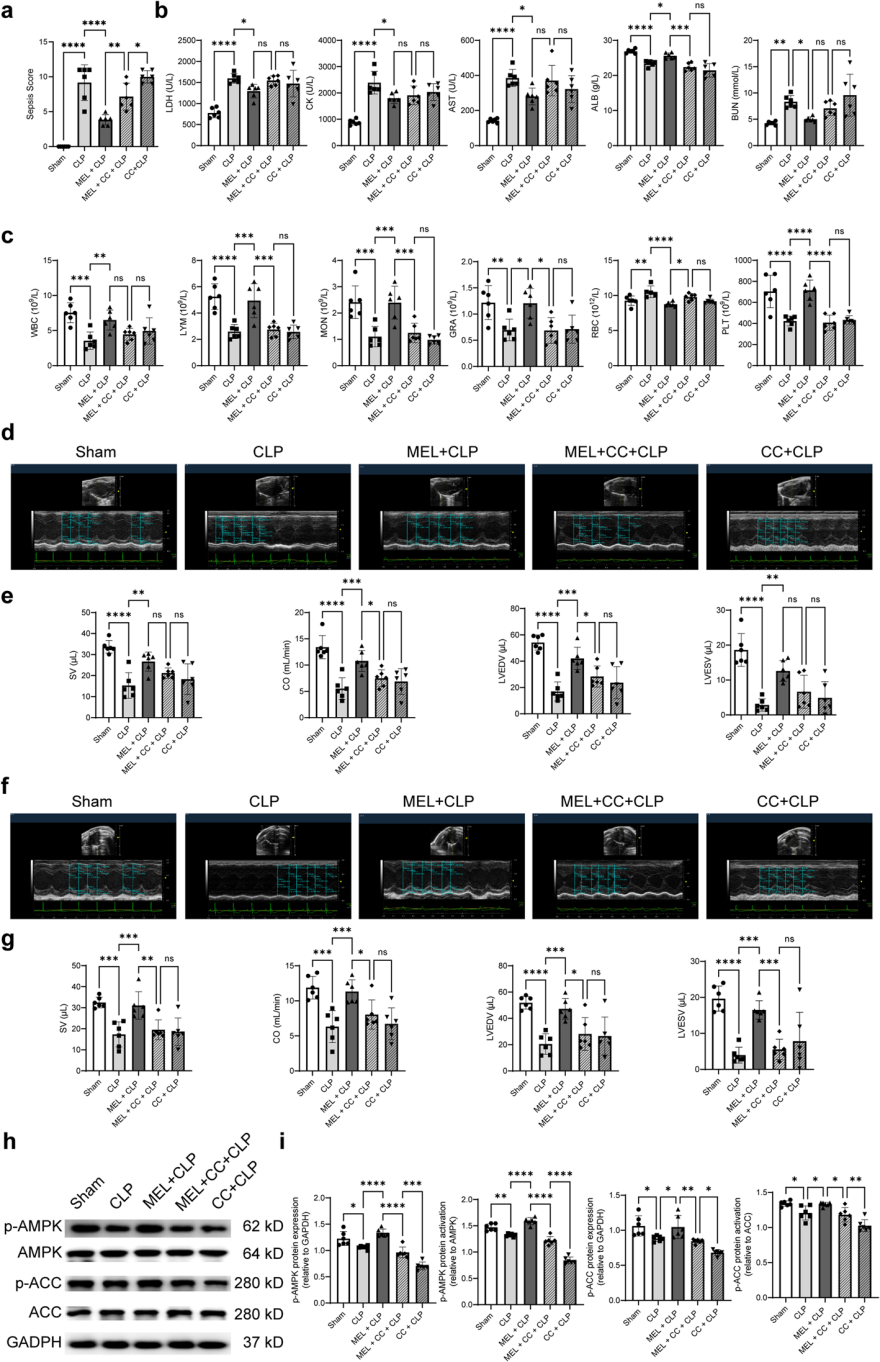
Effect of CC on the protective role of melatonin pretreatment in septic mice. **a** Sepsis scores. **b** Blood biochemical parameters. **c** Blood routine parameters. **d** Representative echocardiography images of the long axis. **e** SV, CO, LVEDV, and LVESV statistical graphs of the long axis. **f** Representative echocardiography images of the short axis. **g** SV, CO, LVEDV, and LVESV statistical graphs of the short axis. **h** Representative western blot protein bands of p-AMPK, AMPK, p-ACC, and ACC in mouse heart tissues. **i** Quantitative analysis of western blot by normalizing to GAPDH. *P < 0.05, **P < 0.01, ***P < 0.001, ****P < 0.0001 versus sham or versus CLP or MEL + CLP; *ns,* nonsignificant. *n* = 6 for each group. Statistical analysis of data was performed using ANOVA

The above study found that CLP injury resulted in markedly decreased phosphorylation levels of AMPK and ACC, while melatonin restored those levels. To further confirm this point, mice were pretreated with Compound C (CC, AMPK inhibitor) and melatonin. As shown in Fig. 6h, i, CC abolished the melatonin-induced phosphorylation levels of AMPK and ACC. CC also reversed the melatonin-induced decrease of the sepsis score in CLP mice (Fig. 6a) and blocked the protective effect of melatonin on blood biochemical and blood routine parameters, as evidenced by increased RBC and decreased ALB, LYM, MON, GRA, and PLT in the MEL+CC+CLP group (Fig. 6b, c). In particular, CC could weaken the positive effect of melatonin on cardiac function, manifested as decreased SV, CO, LVEDV, and LVESV (Fig. 6d–g). Additional echocardiographic data are available in Additional file 1: Fig. S3a, b. Notably, though CC could abolish the protective role of melatonin, but not all the effects were inhibited. Nonetheless, these results confirmed that AMPK plays a key role in melatonin protection against septic myocardial injury.

### RNA-seq results of septic myocardial injury in mice

To fully understand the pathological regulatory mechanisms of melatonin on CLP injury, myocardial transcriptome sequencing and analysis was performed. As shown in Additional file 1: Fig. S4a, principal component analysis (PCA) analysis showed that there were three different transcriptome patterns of sham, CLP, and MEL + CLP group, and each group was clustered well with high sample reproducibility. Among them, the CLP group and the MEL + CLP group showed distinct performances.

**Fig. 7:**
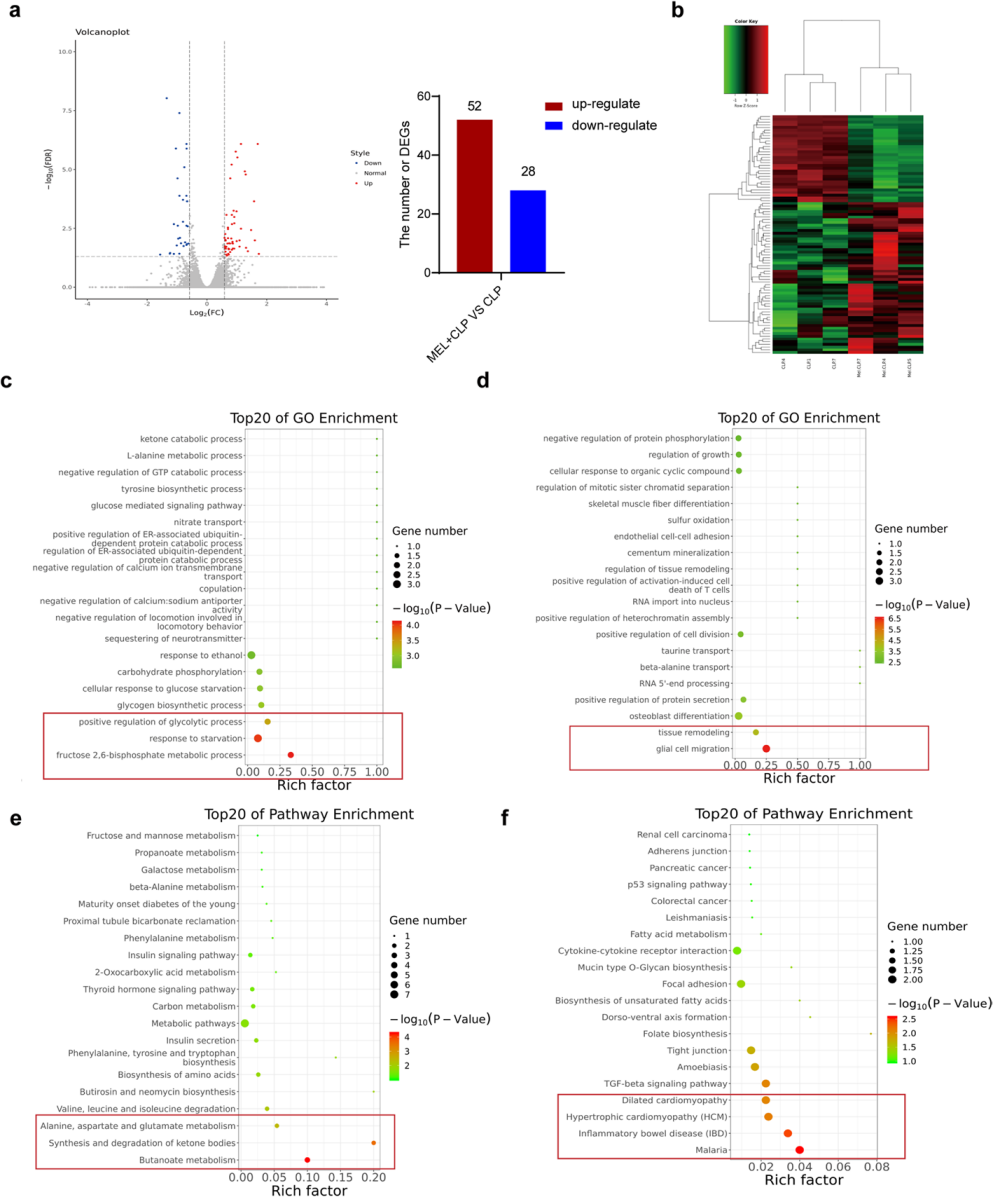
RNA-seq results of septic myocardial injury in mice. **a** Differentially expressed mRNAs are displayed by volcano plot. The blue and red parts indicate a more than twofold decreased and increased expression of the dysregulated mRNAs in cardiac tissues, respectively (*P* < 0.05). **b** Cluster analysis of differential genes in each group. **c** Gene Ontology (GO) analysis of the upregulated gene categories between the CLP group and the MEL + CLP group (*P* < 0.05). **d** GO analysis of the downregulated gene categories between the CLP group and the MEL + CLP group (*P* < 0.05). **e** KEGG pathway analysis of upregulated pathways between the CLP group and the MEL + CLP group (*P* < 0.05). **f** KEGG pathway analysis of downregulated pathways between the CLP group and the MEL + CLP group (*P* < 0.05)

The volcano map and heat map analysis was shown in Fig. 7a, b. Compared with the CLP group, the MEL + CLP group showed a series of significantly different genes. Specifically, 52 genes were upregulated and 28 genes were downregulated, indicating that these genes might be involved in the pathological processes of melatonin against septic myocardial injury. As shown in Additional file 1: Fig. S4b, there were two patterns of genes, respectively: pattern 6 showed that the expression of 578 genes was upregulated and then unchanged; pattern 1 showed that the expression of 306 genes was downregulated and then unchanged.

In addition, GO analysis was performed to determine the functional changes between the CLP group and MEL+CLP group. The upregulated differential genes in the MEL + CLP group were involved in regulating the these biological processes, including metabolic process of fructose 2,6-diphosphate, the response to starvation, and the positive regulation of glycolysis (Fig. 7c and Additional file 1: Fig. S4c). Downregulated differential genes in the MEL + CLP group were involved in biological processes of glial cell migration and tissue remodeling (Fig. 7d and Additional file 1: Fig. S4d).

KEGG analysis further revealed that melatonin might upregulate pathways pertinent to butanoate metabolism, synthesis and degradation of ketone bodies and alanine, and aspartate and glutamate metabolism (Fig. 7e and Additional file 1: Fig. S4e), and simultaneously downregulate pathways pertinent to malaria, inflammatory bowel disease, hypertrophic cardiomyopathy, and dilated cardiomyopathy, as well as the TGF-β signaling pathway (Fig. 7f, Additional file 1: Fig. S4f). Additional data of the transcriptomic analysis in the sham and CLP group are shown in Additional file 1: Fig. S5. Taken together, our study further proved that melatonin had a significant effect on the transcriptome of septic myocardial injury.

### Protective effect of melatonin posttreatment on sepsis and septic myocardial injury in mice

**Fig. 8:**
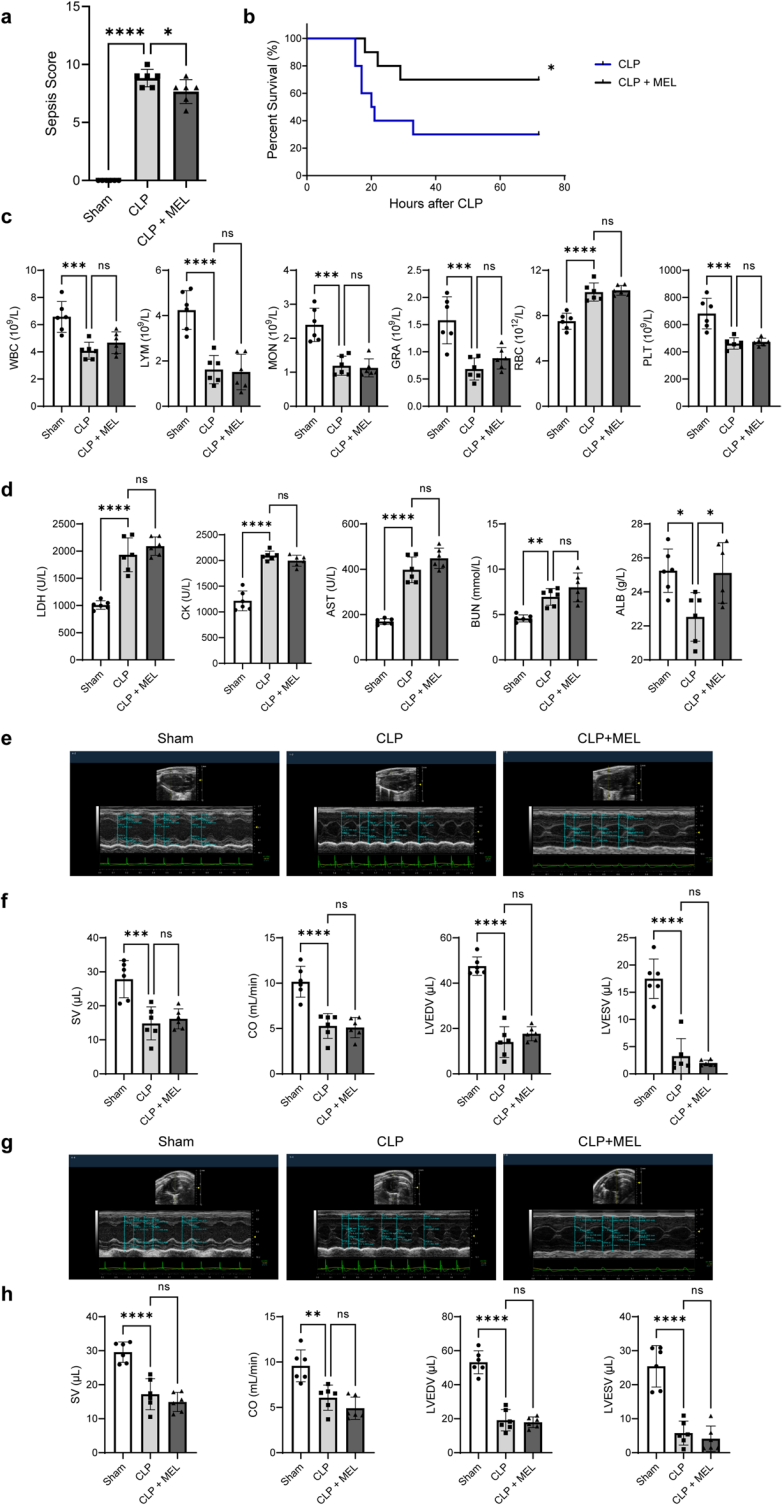
Protective effect of melatonin posttreatment on sepsis and septic myocardial injury in mice. **a** Sepsis scores. **b** Kaplan–Meier survival curves. Ten animals for each group were used for comparison. Mortality was observed within 72 h. **c** Blood routine parameters. **d** Blood biochemical parameters. **e** Representative echocardiography images of the long axis. **f** SV, CO, LVEDV, and LVESV statistical graphs of the long axis. **g** Representative echocardiography images of the short axis. **h** SV, CO, LVEDV, and LVESV statistical graphs of the short axis. *P < 0.05, **P < 0.01, ***P < 0.001, ****P < 0.0001 versus sham or versus CLP; *ns,* nonsignificant. *n* = 6 for each group. Statistical analysis of data was performed using ANOVA

The above data demonstrated that melatonin pretreatment could mitigate septic myocardial injury. Then, the therapeutic potential of melatonin posttreatment was examined in CLP-injured mice. As shown in Fig. 8a, b, melatonin decreased the sepsis score while increased the survival rate to nearly 40% within 72 h after CLP. In addition, though melatonin posttreatment showed no positive effects on other blood biochemical and blood routine parameters, or echocardiographic data, ALB was markedly increased in the CLP + MEL group (Fig. 8c–h and Additional file 1: Fig. S6). These results suggested that melatonin posttreatment had a certain protective effect, but it was not as prominent as pretreatment.

### The synergistic protective effect of melatonin and classical antibiotics on sepsis and septic myocardial injury

**Fig. 9:**
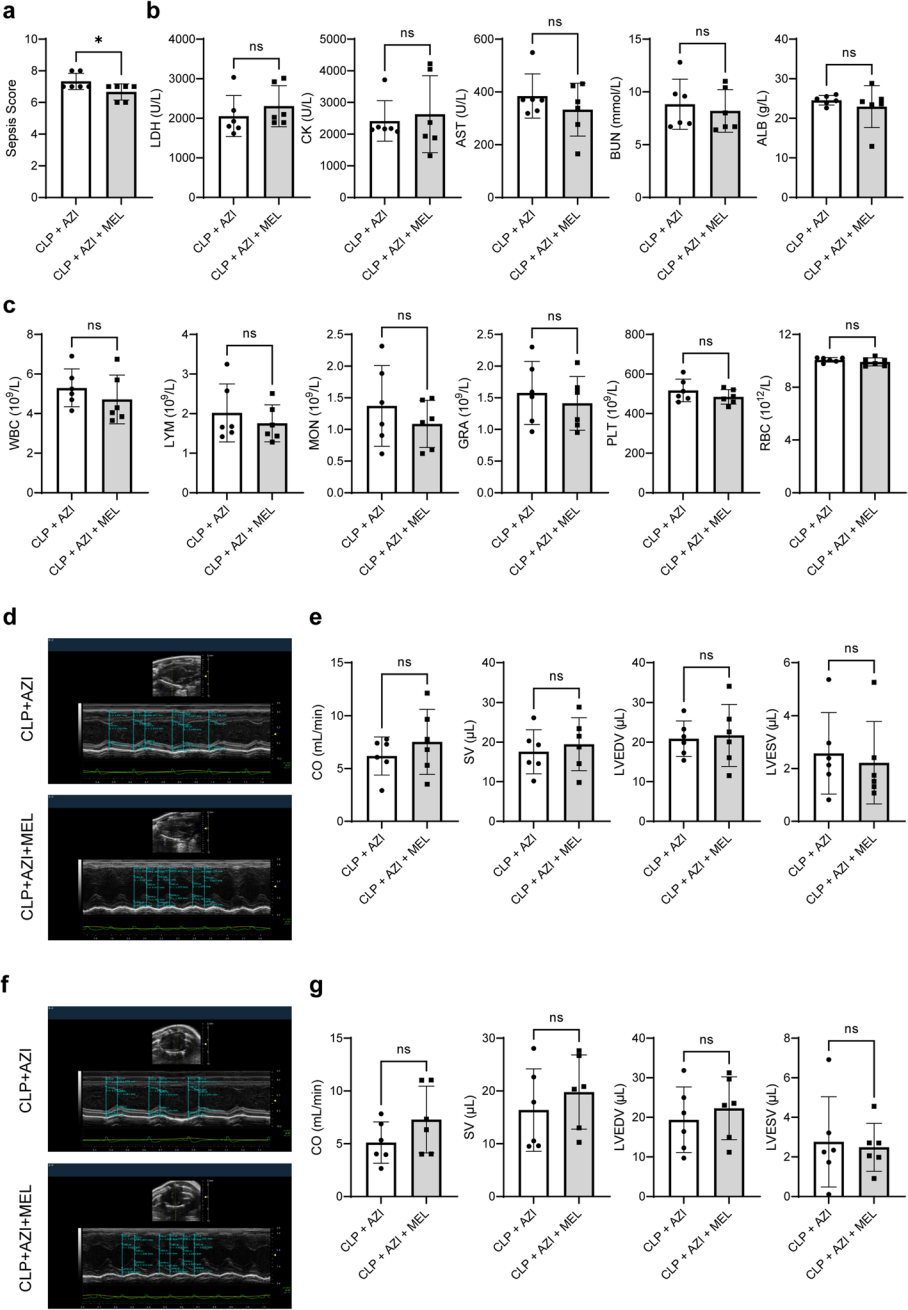
Synergistic protective effect of melatonin and AZI on sepsis and septic myocardial injury in mice. **a** Sepsis scores. **b** Blood biochemical parameters. **c** Blood routine parameters. **d** Representative echocardiography images of the long axis. **e** CO, SV, LVEDV, and LVESV statistical graphs of the long axis. **f** Representative echocardiography images of the short axis. **g** CO, SV, LVEDV, and LVESV statistical graphs of the short axis. ^*^P < 0.05, **P < 0.01, ***P < 0.001, ****P < 0.0001 versus CLP + AZI; *ns,* nonsignificant. *n* = 6 for each group. Statistical analysis of data was performed using *t*-test

**Fig. 10:**
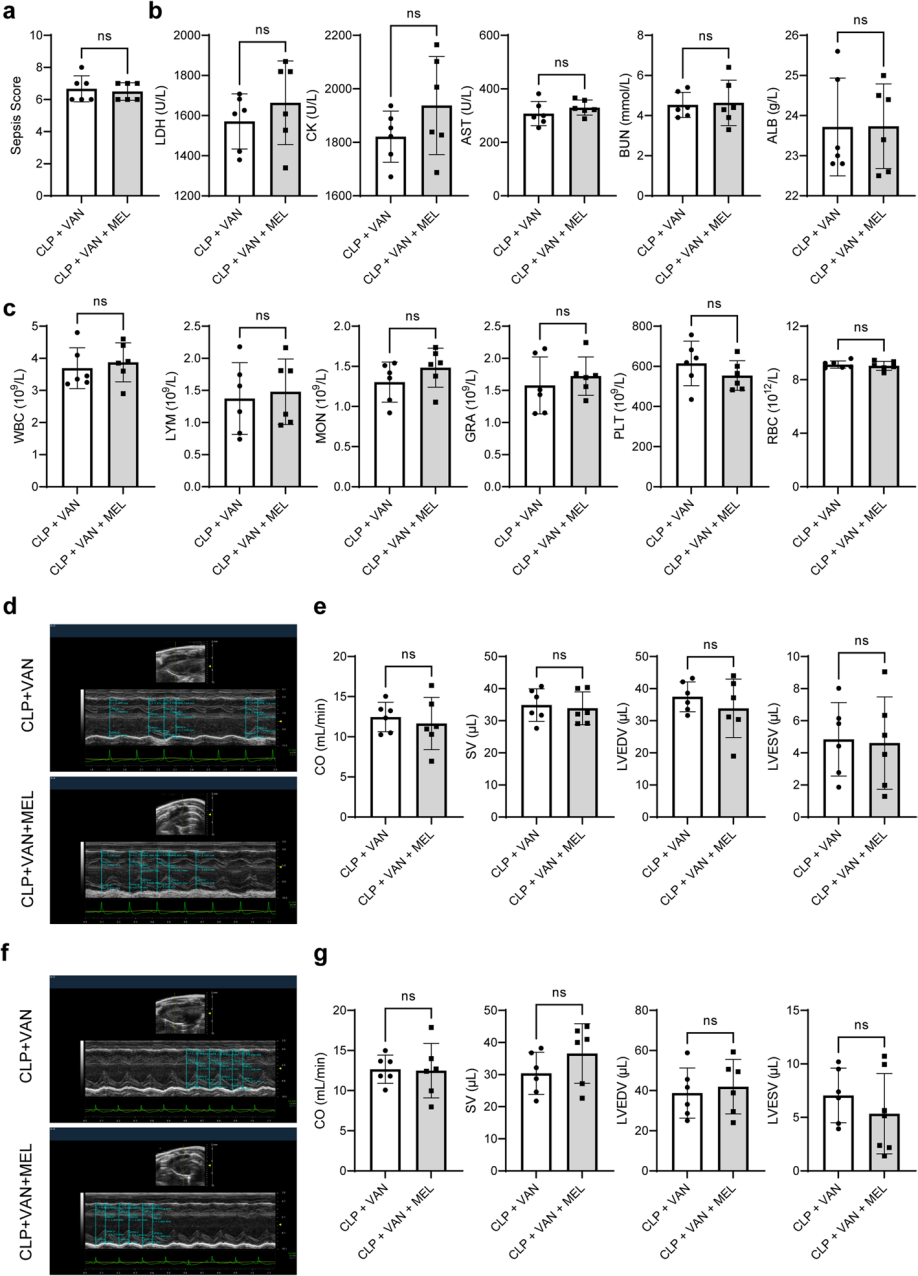
Synergistic protective effect of melatonin and vancomycin on sepsis and septic myocardial injury in mice. **a** Sepsis scores. **b** Blood biochemical parameters. **c** Blood routine parameters. **d** Representative echocardiography images of the long axis. **e** CO, SV, LVEDV, and LVESV statistical graphs of the long axis. **f** Representative echocardiography images of the short axis. **g** CO, SV, LVEDV, and LVESV statistical graphs of the short axis. ^*^P < 0.05, **P < 0.01, ***P < 0.001, ****P < 0.0001 versus CLP + VAN; *ns,* nonsignificant. *n* = 6 for each group. Statistical analysis of data was performed using *t*-test

Clinically, azithromycin and vancomycin are the traditional antibiotics in sepsis treatment [18, 19] To evaluate the synergistic effect of melatonin combined with azithromycin, the role of azithromycin posttreatment in septic myocardial injury was observed. Azithromycin markedly reduced sepsis score (Additional file 1: Fig. S7a), increased survival (Additional file 1: Fig. S7b), and improved blood routine parameters (increased GRA, Supplementary Fig. S7c), blood biochemical parameters (reduced LDH and BUN, Additional file 1: Fig. S7d), and cardiac function parameters (increased LVEDV and decreased HR, Supplementary Fig. S7e–h) of CLP mice. Then, the effect of melatonin combined with azithromycin was evaluated. Compared with the CLP + AZI group, melatonin combined with azithromycin could remarkably reduce sepsis score (Fig. 9a), while have no significant effect on blood biochemical, blood routine, and cardiac function parameters in septic mice (Fig. 9b–g and Additional file 1: Fig. S8a, b). To further consolidate the results, the synergistic effect of melatonin and another classical antibiotic, vancomycin, was observed. Similarly, vancomycin showed significant protective effect, manifested as the reduced sepsis score (Supplementary Fig. S9a), improved blood routine and blood biochemical parameters (increased PLT and decreased LDH, AST, and BUN, Additional file 1: Fig. S9c, d), and improved cardiac function parameters (increased SV, CO, LVEDV and LVESV, and reduced LVPWd and LVPWs) (Additional file 1: Fig. S9e–h), though vancomycin showed no effect on anal temperature (Additional file 1: Fig. S9b). However, the effect of melatonin combined with vancomycin showed no significant change in CLP mice (Fig. 10a–g and Additional file 1: Fig. S8c, d). These data indicated that the melatonin and azithromycin/vancomycin co-administration displayed a limited synergistic effect against septic myocardial injury.

## Discussion

Despite recent advances in sepsis, there are still few effective treatments available beyond the standard of care and supportive therapy. Treatment of septic heart failure remains a huge challenge for clinicians and basic researchers. Melatonin is a multifunctional endogenous indoleamine molecule that participates in the regulation of oxidative stress, inflammation, autophagy, and ERS [20, 21]. On the basis of the various characteristics of melatonin, it has been reported that melatonin shows a protective effect on organ dysfunction in experimental models of sepsis. Chen et al. proved that, in CLP-induced rats, melatonin posttreatment efficiently attenuated liver dysfunction possibly by targeting SIRT1/STAT3 pathway in the liver [22]. As evidenced in this study, melatonin pretreatment could improve the survival rate of mice, reduce the sepsis score, and increase anal temperature. In addition, melatonin also reversed the increase in serum biomarkers (LDH, CK, AST, and BUN) and the decrease in cardiac function indicators (SV, CO, LVEDV, and LVESV) induced by CLP injury, showing the powerful cardioprotective effect of melatonin.

AMPK, an evolutionarily conserved energy sensor, plays a crucial role in various diseases, including inflammation [23], diabetes [24], and cancer [25]. The AMPK-dependent mechanism is also considered as a potential therapeutic target of sepsis. The absence of AMPK in bone marrow cells increases the release of high-mobility group box 1 (HMGB1, a late mediator of inflammation), which in turn leads to the occurrence of sepsis in mice [26]. Importantly, AMPK participates in the pathological process of septic myocardial injury by targeting downstream target ACC. Sun et al. demonstrated that leukotriene B4 receptor type 1 antagonist attenuates LPS-induced acute cardiac dysfunction via the activation of the AMPK/ACC signaling pathway [27]. Further study found that AMPK inhibitor CC significantly reverses the protection of the BLT1 antagonist against cardiac dysfunction [27]. Pharmacological activation of AMPK may represent a cardioprotective strategy for the treatment of sepsis and septic myocardial injury. Our study showed that CLP injury significantly decreased phosphorylation levels of AMPK and ACC, which were reversed by melatonin pretreatment. These results demonstrated the key role of AMPK in septic myocardial injury. Further studies have shown that CC reversed the myocardial protective effects of melatonin. Compound C is a classical AMPK inhibitor that has been applied in a variety of cardiovascular studies [28, 29]. We found that though CC could abolish the protective role of melatonin, not all the effects were inhibited. This may be explained by the fact that CC is a small molecule compound and has pleiotropic functions, which may also affect other signaling pathways and have AMPK-independent activities. Nonetheless, these results exactly confirmed that AMPK plays a key role in melatonin protection against septic myocardial injury. Interestingly, another study showed that AMPK activity and the phosphorylation level of ACC increase in the early stage of sepsis [10]. The difference may be related to the detection timepoint and the degree of injury, as severe injury may result in an inappropriate adaptive reaction.

Sepsis-induced hyperinflammatory state is the combined actions for pathogen-associated molecular patterns (PAMPs) and damage-associated molecular patterns (DAMPs). PAMPs and DAMPs recognized by pattern recognition receptor (PRR) initiate an overproduction of pro-inflammatory cytokines (TNF-α, HMGB1, etc.) and chemokines (CXCL2, etc.) [30]. It is meaningful to treat septic myocardial injury by focusing on the key anti-inflammatory targets. Previous research has established the anti-inflammatory role of melatonin in organ damage caused by sepsis. In LPS rats, increased levels of TNF-α, IL-10, and NF-κB in plasma were observed, whereas these alterations were attenuated by melatonin pretreatment [31]. Besides, melatonin pretreatment disrupts NF-κB transcriptional activity and NF-κB/NLRP3 connection in septic mice, suggesting potent anti-inflammatory effects of melatonin against systemic innate immune activation [32]. This study also verified that melatonin pretreatment alleviates inflammation, as shown by significant reduction of inflammatory markers and pro-inflammatory factors. The work further confirmed the powerful anti-inflammatory ability of melatonin against sepsis and septic myocardial injury.

During septic heart failure, the oxidative and antioxidant systems are thrown out of balance, leading to excessive production of ROS [33]. Excessive ROS impairs the function of mitochondria and leads to disturbance of cardiac energy metabolism. Therefore, stimulating the endogenous antioxidant system provides an entry point to developing drugs for septic myocardial injury. Previous studies have shown that melatonin has significant antioxidative activity and can increase the level of antioxidant enzymes and effectively scavenge free radicals [34]. In this regard, the results also found that CLP induced severe oxidative stress as shown by increased DHE signals, whereas melatonin reduced this level. To further clarify the molecular mechanism of melatonin against oxidative stress in sepsis, we also focused on the antioxidant targets, including Nrf2 and HO-1. Studies reported that Nrf2 translocates from the cytoplasm to the nucleus and activates nuclear antioxidant enzymes HO-1, both of which are involved in the antioxidant effect of Nrf2 [35]. In this study, melatonin could promote the increase of Nrf2 and HO-1, suggesting that these molecules may be the key regulators of melatonin in antioxidative stress against septic myocardial injury.

Cardiac dysfunction caused by sepsis is often manifested as weakened systolic function, which is closely associated with mitochondrial dysfunction [36]. Decreased activity of mitochondrial respiratory chain complex I and the sudden decrease in ATP synthesis are correlated with the severity and outcome of septic heart failure [37]. Therefore, activation of mitochondrial biogenesis or improvement of mitochondrial function is a protective strategy to reduce cardiomyocyte vulnerability during sepsis. COXIV and SDH5 can provide electrons for the mitochondrial respiratory chain to participate in the respiratory function of mitochondria [38, 39]. Mitochondrial biogenesis is regulated by several genes, such as *NRF1*, *TFAM*, and *UCP2*, to control mitochondrial synthesis and protein expression. NRF1 coordinates the stress-inducible activation of a vast cytoprotective genes, including TFAM [40]. Studies have shown that LPS-induced sepsis results in upregulation of NRF1 and TFAM, which in turn stimulates mtDNA replication and mitochondrial biogenesis [41]. UCP2 is a carrier protein located on the inner mitochondrial membrane. Knocking out UCP2 aggravates the destruction of mitochondrial ultrastructure and causes more severe edema to the mitochondrial inner membrane under septic conditions, hinting the positive role of UCP2 in sepsis [42]. As expected, the results from this study showed that CLP injury also suppressed TFAM, UCP2, COXIV, SDH5, and NRF1, while melatonin pretreatment restored the expression of these proteins, which also contributed to the protective roles of melatonin against septic myocardial injury.

Organ failure caused by sepsis is also closely related to ERS. ERS can trigger a series of adaptive responses by activating the unfolded protein response (UPR) [43]. UPR is mainly mediated by PERK, inositol-requiring enzyme-1 (IRE1), and ATF6. In normal physiological conditions, GRP78 binds to these molecules and inactivates them, where GRP78 can activate UPR once separated from these molecules to restore homeostasis of the physiological function in the endoplasmic reticulum. Otherwise, once the UPR cascade is insufficient to restore the ERS, apoptosis would be initiated [44]. As important markers of ERS, these molecules are considered to be essential mediators of sepsis. In the LPS-injured myocardial tissues, the expression of GRP78, PERK, and IRE1α increased, suggesting the contribution of ERS in the heart [45]. Astrid et al. proved that melatonin posttreatment modifies cellular stress in the liver via upregulation of PERK and CHOP in severe sepsis [46]. Our study showed increased levels of CHOP, GRP78, and ATF6, but reduced p-PERK protein level in the MEL + CLP group. The results were not completely consistent with those of previous studies, whichmight be due to the detection time point and the damage degree. Nonetheless, the data fully illustrated that the cardioprotection of melatonin is dependent on the regulation of ERS.

Sepsis is an abnormally amplified inflammatory response, and fibrosis is often a consequence of inflammation [47, 48]. In this study, hematoxylin–eosin (H&E) staining and Masson staining showed there was myocardial fibrosis in the heart of CLP mice, whereas melatonin could reverse the changes induced by CLP. Transcriptome sequencing was performed to further clarify the myocardial protective mechanism of melatonin. The myocardial protective mechanism of melatonin was involved in various signaling pathways, including the downregulated pathways pertinent to fibrosis (glial cell migration and tissue remodeling, fibronectin binding and actin binding, and extracellular matrix and extracellular space), which also strongly proved the antifibrotic effect of melatonin in septic myocardial injury. In future research, we will further verify the signaling pathways obtained by RNA-seq and clarify the specific mechanisms of the biological processes.

Clinically, patients usually start taking drugs after injury occurs, hence, pharmacological posttreatment is more meaningful in clinical application. This study also focused on the therapeutic potential of melatonin in septic mice. The results showed that melatonin posttreatment exhibited a certain degree protective effect. However, overall, the pre-protection effect of melatonin is better than the post-protection effect. This might be explained by the fact that melatonin has given mice armor-like protection in advance in the pretreatment experiments. In contrast, if melatonin administration were performed after CLP injury, the protective effect would be weakened because of the severe CLP injury.

Azithromycin and vancomycin are commonly used antibiotics for the treatment of mycoplasma pneumoniae infections [49], bacterial infections [50], and chronic inflammatory diseases [51]. The significant anti-inflammatory effect of azithromycin in sepsis has also been widely recognized, especially in combination with other antibiotics. Azithromycin pretreatment can reduce the plasma TNF-α level and increase the survival rate of mice induced by LPS [52]. Notably, the combination of melatonin and ampicillin plus gentamicin can improve the clinical outcome of neonatal sepsis [53]. Therefore, in addition to focusing on the protection of melatonin alone, the study also explored whether the combination of melatonin and azithromycin (or vancomycin) showed a synergistic protective effect. First, the role of azithromycin posttreatment in septic myocardial injury was observed. The results showed that azithromycin posttreatment had cardioprotection, indicted by reduced sepsis score, increased the survival rate, and improved blood routine parameters and blood biochemical parameters as well as cardiac function parameters, though not all parameters were modified. This might be due to the fact that the protective effect of azithromycin was a post-protection, which was essentially different from pre-protection. Therefore, compared with the pre-protection of melatonin, the post-protection of azithromycin was slightly limited but better than the post-protection of melatonin, which also provided a strong rationale that azithromycin is a classical anti-infective drug commonly used in clinic. According to clinical medication specifications, azithromycin is an antibiotic and cannot be used as a preventive and protective medicine before infective injury. However, melatonin is a commonly used healthcare product for the treatment of insomnia, which is feasible as a preventive medicine. These two strategies have their own advantages and drawbacks, and we can utilize the advantages of these two therapies in the treatment of sepsis. In addition, the combination therapy results in this study showed that melatonin and azithromycin or vancomycin improved only the sepsis score and some indicators. We speculated that, on the one hand, antibiotics have a significant therapeutic effect on sepsis, and cannot highlight the synergistic effect of melatonin combined administration. On the other hand, azithromycin is a biphasic time- and phase-dependent immunomodulatory action, and its effectiveness decreases with time and sepsis severity. Likewise, as melatonin is obtained from an exogenous source, its level is related to the circadian rhythm and may be different at different times.

## Conclusions

**Fig. 11:**
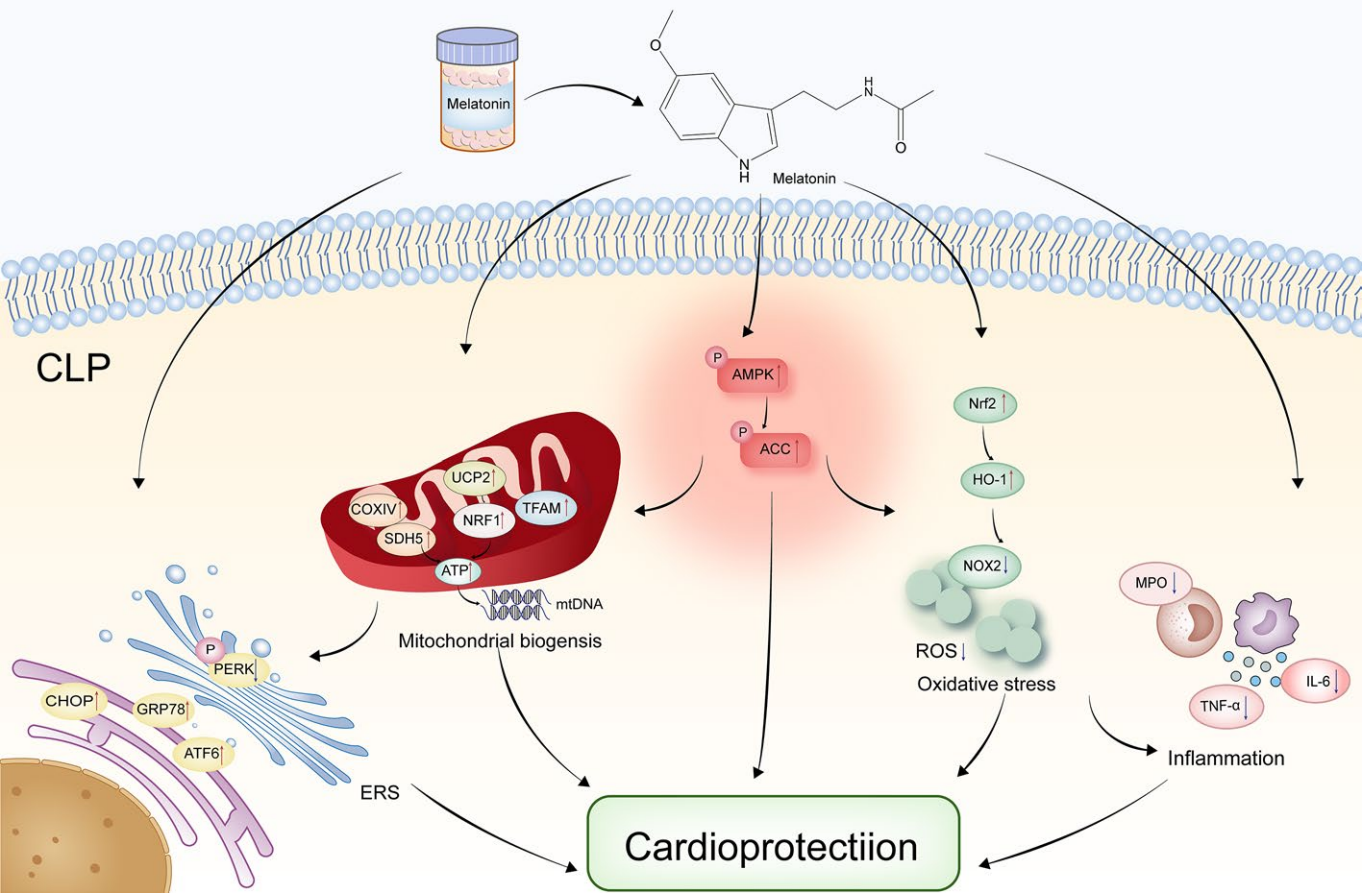
Proposed scheme depicting the mechanisms by which melatonin pretreatment protects against sepsis and septic myocardial injury

Taken together, the current study evaluated the different administration methods of melatonin and the therapeutic potential of melatonin in combination with classical antibiotics. According to the study, melatonin pretreatment confers a significant protective effect against septic heart failure, which contributed to attenuation of inflammation and oxidative stress, improvement of mitochondrial function, and regulation of ERS, together with activation of the AMPK signaling pathway (Fig. 11). Melatonin posttreatment has a certain therapeutic effect, but the effect is not as significant as pretreatment. In addition, the combination of melatonin and classical antibiotics has a slight synergistic protective effect. This work makes a more detailed examination of the potential clinical use of melatonin in treating septic myocardial injury.

## Supplementary Information

**Additional file 1:** **Fig. S1.** Establishment of mouse CLP (**a**) and aggravated CLP models (**b**). The cecum was tightly ligated at 1/3 site from its end using 4–0 nylon suture, and double punctures of the cecal wall were performed with a 25 G needle. For the aggravated CLP model, the cecum was tightly ligated at 2/3 site from its end. **Fig. S2.** Additional echocardiographic data of melatonin pretreatment in septic mice. **a** LVPWd, LVPWs, HR, and LV mass statistical graphs of the long axis. **b** LVPWd, LVPWs, HR, and LV mass statistical graphs of the short axis. *P < 0.05, **P < 0.01, ***P < 0.001, ****P < 0.0001 versus Sham or versus CLP or MEL + CLP; *ns* nonsignificant. *n* = 6 for each group. Statistical analysis of data was performed using ANOVA. **Fig. S3.** Additional echocardiographic data on the effect of Compound C for melatonin pretreatment in septic mice. **a** LVPWd, LVPWs, HR, and LV mass statistical graphs of the long axis. **b** LVPWd, LVPWs, HR, and LV mass statistical graphs of the short axis. *P < 0.05, **P < 0.01, ***P < 0.001, ****P < 0.0001 versus Sham or versus CLP or MEL + CLP; *ns* non-significant. *n* = 6 for each group. Statistical analysis of data was performed using ANOVA. **Fig. S4.** Additional data of RNA sequencing between the CLP group and the MEL + CLP group. **a** PCA diagram of each group. **b** Expression profiles in color indicating significant ones (*P* < 0.05). Red indicates upregulated and green indicates downregulated. Profile number (top left), gene number (bottom left), and trend (line) in each profile are also labeled. **c** GO analysis of the upregulated gene categories (*P* < 0.05). **d** GO analysis of the downregulated gene categories. **e** KEGG pathway analysis of upregulated pathways (*P* < 0.05). **f** KEGG pathway analysis of downregulated pathways (*P* < 0.05). **Fig. S5.** Additional data of RNA sequencing between the sham group and the CLP group. **a** Differentially expressed mRNAs were displayed by volcano plot. The blue and red parts indicate more than twofold decreased and increased expression of the dysregulated mRNAs in cardiac tissues, respectively (*P* < 0.05). **b** Cluster analysis of differential genes in the sham group and the CLP group. **c** GO analysis of the upregulated gene categories between the sham group and the CLP group. (*P* < 0.05). **d** GO analysis of the downregulated gene categories between the sham group and the CLP group (*P* < 0.05). **e** KEGG pathway analysis of upregulated pathways between the sham group and the CLP group (*P* < 0.05). **f** KEGG pathway analysis of downregulated pathways between the sham group and the CLP group (*P* < 0.05). **Fig. S6. **Additional echocardiographic data of melatonin posttreatment in septic mice. **a** LVPWd, LVPWs, HR, and LV mass statistical graphs of the long axis. **b** LVPWd, LVPWs, HR, and LV mass statistical graphs of the short axis. *P < 0.05, **P < 0.01, ***P < 0.001, ****P < 0.0001 versus Sham or versus CLP ; *ns* nonsignificant. *n* = 6 for each group. Statistical analysis of data was performed using ANOVA. **Fig. S7.** Protective effect of azithromycin against CLP-induced myocardial injury in mice. **a** Sepsis scores. **b** Kaplan–Meier survival curves. Ten animals for each group were used for comparison. Mortality was observed within 72 h. **c** Blood routine parameters. **d** Blood biochemical parameters. **e** Representative echocardiography images of the long axis. **f** SV, CO, LVEDV, LVESV, LVPWd, LVPWs, HR, and LV mass statistical graphs of the long axis. **g** Representative echocardiography images of the short axis. **h** SV, CO, LVEDV, LVESV, LVPWd, LVPWs, HR, and LV mass statistical graphs of the short axis. *P < 0.05, **P < 0.01, ***P < 0.001, ****P < 0.0001 versus Sham or versus CLP; *ns* nonsignificant. *n* = 6 for each group. Statistical analysis of data was performed using ANOVA. **Fig. S8. **Additional echocardiographic data of melatonin combined with azithromycin or vancomycin in septic mice. **a** LVPWd and LV mass statistical graphs of the long axis. **b** LVPWd and LV mass statistical graphs of the short axis. **c** LVPWd and LV mass statistical graphs of the long axis. **d** LVPWdand LV mass statistical graphs of the short axis. *P < 0.05, **P < 0.01, ***P < 0.001, ****P < 0.0001 versus CLP+AZI or versus CLP + VAN; *ns* nonsignificant. *n* = 6 for each group. Statistical analysis of data was performed using *t*-test. **Fig. S9. **Protective effect of vancomycin against CLP-induced myocardial injury in mice. **a** Sepsis scores. **b** Anal temperature. **c** Blood routine parameters. **d** Blood biochemical parameters. **e** Representative echocardiography images of the long axis. **f** SV, CO, LVEDV, LVESV, LVPWd, LVPWs, HR, and LV mass statistical graphs of the long axis. **g** Representative echocardiography images of the short axis. **h** SV, CO, LVEDV, LVESV, LVPWd, LVPWs, HR, and LV mass statistical graphs of the short axis. *P < 0.05, **P < 0.01, ***P < 0.001, ****P < 0.0001 versus Sham or versus CLP; *ns* nonsignificant. *n* = 6 for each group. Statistical analysis of data was performed using AVONA. Additional materials and methods section and table.

## Data Availability

The datasets used and/or analysis during the current study are available from the corresponding author on reasonable request.

## Abbreviations

AMPK: AMP-activated protein kinase
LPS: Lipopolysaccharide
CLP: Cecal ligation and puncture
MSS: Murine sepsis score
WBC: White blood cells
LYM: Lymphocytes
MON: Monocyte
GRA: Granulocytes
PLT: Platelets
ALB: Albumin
RBC: Red blood cells
LDH: Lactic dehydrogenase
CK: Creatine kinase
AST: Aspartate aminotransferase
BUN: Blood urea nitrogen
SV: Stroke volume
CO: Cardiac output
LVEDV: Left ventricular diastolic volume
LVESV: Left ventricular systolic volume
ROS: Reactive oxygen species
ERS: Endoplasmic reticulum stress
IHC: Immunohistochemistry
PCA: Principal component analysis
HMGB1: High-mobility group box 1
PAMPs: Pathogen-associated molecular patterns
DAMPs: Damage-associated molecular patterns
PRR: Pattern recognition receptor
UPR: Unfolded protein response
IRE1: Inositol-requiring enzyme-1
H&E: Hematoxylin–eosin
