# Supplementary material for: Protection of melatonin treatment and combination with traditional antibiotics against septic myocardial injury
Source: Cell Mol Biol Lett. 2023 Apr 26;28:35. doi: 10.1186/s11658-022-00415-8 (PMC10134561; doi:10.1186/s11658-022-00415-8)
Supplement: Supplementary file 1 — Additional file 1: Fig. S1. Establishment of mouse CLP (a) and aggravated CLP models (b). The cecum was tightly ligated at 1/3 site from its end using 4–0 nylon suture, and double punctures of the cecal wall were performed with a 25 G needle. For the aggravated CLP model, the cecum was tightly ligated at 2/3 site from its end. Fig. S2. Additional echocardiographic data of melatonin pretreatment in septic mice. a LVPWd, LVPWs, HR, and LV mass statistical graphs of the long axis. b LVPWd, LVPWs, HR, and LV mass statistical graphs of the short axis. *P < 0.05, **P < 0.01, ***P < 0.001, ****P < 0.0001 versus Sham or versus CLP or MEL + CLP; ns nonsignificant. n = 6 for each group. Statistical analysis of data was performed using ANOVA. Fig. S3. Additional echocardiographic data on the effect of Compound C for melatonin pretreatment in septic mice. a LVPWd, LVPWs, HR, and LV mass statistical graphs of the long axis. b LVPWd, LVPWs, HR, and LV mass statistical graphs of the short axis. *P < 0.05, **P < 0.01, ***P < 0.001, ****P < 0.0001 versus Sham or versus CLP or MEL + CLP; ns non-significant. n = 6 for each group. Statistical analysis of data was performed using ANOVA. Fig. S4. Additional data of RNA sequencing between the CLP group and the MEL + CLP group. a PCA diagram of each group. b Expression profiles in color indicating significant ones (P < 0.05). Red indicates upregulated and green indicates downregulated. Profile number (top left), gene number (bottom left), and trend (line) in each profile are also labeled. c GO analysis of the upregulated gene categories (P < 0.05). d GO analysis of the downregulated gene categories. e KEGG pathway analysis of upregulated pathways (P < 0.05). f KEGG pathway analysis of downregulated pathways (P < 0.05). Fig. S5. Additional data of RNA sequencing between the sham group and the CLP group. a Differentially expressed mRNAs were displayed by volcano plot. The blue and red parts indicate more than twofold decreased and increased [file 11658_2022_415_MOESM1_ESM.docx]

**Protection of melatonin treatment and combination with traditional antibiotics against septic myocardial injury**

**SUPPLEMENTARY MATERIAL**

**Materials and methods**

**Detection of blood routine parameters and blood biochemical parameters**

At 8 h post-CLP, at least 10 μL blood was collected from the left eyeball into a heparin-coated tube. The levels of blood routine parameters, including white blood cells (WBC), monocyte (MON), lymphocytes (LYM), granulocytes (GRA), red blood cells (RBC), platelets (PLT) were detected by an automatic blood analyzer (Genrui Technology Co., Ltd, KT6200VET, Shenzhen, Guangdong, China). Then, 150 μL serum was isolated from the rest of the whole blood by being centrifuged at 3 000 rpm for 10 min. The levels of blood biochemical parameters, including lactic dehydrogenase (LDH), creatine kinase (CK), aspartate aminotransferase (AST), albumin (ALB), and blood urea nitrogen (BUN) were detected by an automatic blood biochemical analyzer (XinRui Technology Co., Ltd, XR210, Zhongshan, Guangdong, China).

**Echocardiography**

Animal-specific instrument (VisualSonics Vevo3100, VisualSonics, Toronto, ON, Canada) at 8 h post-CLP was used for transthoracic echocardiography. Stroke volume (SV), cardiac output (CO), left ventricular diastolic volume (LVEDV), left ventricular systolic volume (LVESV), left ventricular posterior wall thickness of systole period (LVPWs), and left ventricular posterior wall thickness of diastole period (LVPWd), were measured using Vevo LAB 3.0.0 software.

**Histological staining and masson staining**

The myocardium was fixed in 4% paraformaldehyde and sectioned at a thickness of 4-5 µm. Morphological changes in myocardium were observed by hematoxylin-eosin (H&E) staining. The degree of myocardial fibrosis was examined by Masson staining (Solarbio, Co., Ltd, Beijing, China). For immunostaining, paraffin-embedded slices were stained with the respective primary antibody against MPO (1:200, Servicebio, Co., Ltd, Wuhan, hubei, China), NOX2 (1:200, Servicebio, Co., Ltd, Wuhan, hubei, China), IL-6 (1:200, Servicebio, Co., Ltd, Wuhan, hubei, China), TNF-α (1:200, Servicebio, Co., Ltd, Wuhan, hubei, China), then incubated with a secondary biotinylated anti-rabbit IgG, stained with 3,3’-diaminobenzidine (DAB), and imaged using a microscope (Invitrogen EVOS M5000, Thermo Fisher Scientific, Waltham, MA, USA). Finally, immunoreactive areas were quantified using the ImagePro Plus 4.5 software (Media Cybernetics, Silver Spring, USA).

**Quantitative real-time PCR (qRT-PCR)**

Total RNA was extracted from tissues using the TRIzolTM total RNA extraction kit (TAKARA BIO INC. Kusatsu, Shiga, Japan), and reverse transcription was performed using the Prime Script RT Master Mix (TAKARA BIO INC. Kusatsu, Shiga, Japan). Then TNF-α, IL-6, IL-8, CXCL2, NLRP3, and IL-1β mRNA level is detected using quantitative real-time reverse transcriptase PCR analyses with SYBR Premix Ex Taq (Hunan Accurate Biotechnology Co. Ltd. Hunan, China). The reaction conditions were as follows: (1) 95℃ for 10 min, (2) 40 cycles of 95℃ for 5 s and 60℃ for 30 s, (3) 94℃ for 30 s, 60℃ for 90 s, 94℃ for 10 s. The expression levels of the examined transcripts were compared to that of β-actin. The primer sets used in this study are shown in Supplementary Table 1.

**Western blot**

Isolated heart tissue was homogenized in RIPA buffer containing protease and phosphatase inhibitors (Beyotime Biotechnology, Shanghai, China). The protein concentration was assessed by Enhanced BCA Protein Assay Kit (Beyotime Biotechnology, Shanghai, China). 30-50μg of total protein extract was applied to 10% (or 8%) SDS-PAGE and transferred onto PVDF membranes. The membranes were blocked with 5% defatted milk and incubated with antibodies against AMPK, HO-1 antibodies (Abcam, Cambridge, United Kingdom); p-ACC, ACC, GRP78 antibodies (Cell Signaling Technology, Inc, USA); PERK,SDH5 antibodies (Proteintech, Rosemont, USA); NLRP3, p-PERK antibodies (Bioss Biotechnology, Co., Ltd, Beijing, China); CHOP, COXIV antibodies (Immunoway, Suzhou, Jiangsu, China); ATF6, TFAM antibodies (Santa Cruz Biotechnology, Dallas, TX, USA); Nrf2 antibodies (Boster Biological Technology co.ltd, Inc.USA); GAPDH, Caspase-1,UCP2 antibodies (Servicebio, Co., Ltd, Wuhan, hubei, China); p-AMPK antibodies (Bimake, Houston TX, USA). The signal was quantified using ImageJ 1.8.0 software.

**Bioinformatics analysis**

Total RNA was extracted from animal tissues by Trizol reagent (Invitrogen Life Technology Co., Ltd, USA) separately. The complementary DNA (cDNA) libraries were prepared using the NEBNext^TM^ Ultra Directional RNA Library Prep Kit, NEBNext Poly（A）mRNA Magnetic Isolation Module, NEBNext Multiplex Oligos according to the manufacturer’s instructions. The products were purified and enriched by PCR to create the final cDNA libraries and quantified by Agilent2200. The tagged cDNA libraries were pooled in equal ratio and used for 150 bp paired-end sequencing in a single lane of the Illumina HiSeqXTen. Before read mapping, clean reads were obtained from the raw reads by removing the adaptor sequences, reads with >5% ambiguous bases (noted as N), and low-quality reads containing more than 20% of bases with qualities of <20. The clean reads were then aligned to the mouse genome (version: mm10 NCBI) using the hisat2[1]. HTSeq-FPKM [2] was used to count gene, and the reads per kilobase per million mapped reads (RPKM) method was used to determine the gene expression. After the significant *P*-value analysis and false discovery rate (FDR) analysis, we applied the EBSeq algorithm [3] to filter the differentially expressed genes based on the criteria introduced in this reference[4]. mRNA filtration was as follows: i) Fold Change>2 or <0.5; ii), FDR<0.05. Gene ontology (GO) analysis was performed to elucidate the biological implications of unique genes in the significant or representative profiles of the target gene of the differentially expressed miRNA in the experiment[5]. Pathway analysis was used to determine the significant pathway of the differential genes based on the Kyoto Encyclopedia of Genes and Genomes (KEGG) database. We turned to Fisher’s exact test to select the significant pathway where *P*<0.05 and FDR<0.05 defined the threshold of significance[6].

**Supplementary Table 1.**

| Name | Sequence (5’-3’) |
| --- | --- |
| TNF-α-F | 5’- ACTGAACTTCGGGGTGATCG -3’ |
| TNF-α-R | 5’- TGGTGGTTTGCTACGACGTG -3’ |
| IL-6-F | 5’- TCCGGAGAGGAGACTTCACA -3’ |
| IL-6-R | 5’- TGCCATTGCACAACTCTTTTCT -3’ |
| IL-8-F | 5’- ATGGCTGCTCAAGGCTGGTC -3’ |
| IL-8-R | 5’- GACCAGCCTTGAGCAGCCAT -3’ |
| CXCL2-F | 5’- CCACCAACCACCAGGCTACA -3’ |
| CXCL2-R | 5’- CTGTAGCCTGGTGGTTGGT -3’ |
| NLRP3-F | 5’- TCTACTCTATCAAGGACAGGAACG -3’ |
| NLRP3-R | 5’- CCTTTCTCGGGCGGGTAAT -3’ |
| IL-1β-F | 5’- CCTTGTGCAAGTGTCTGAAGC -3’ |
| IL-1β-R | 5’- AAGGGCTTGGAAGCAATCCT -3’ |
| Caspase1-F | 5’- AGAACAGAACAAAGAAGATGGCACA -3’ |
| Caspase1-R | 5’- GTGCCATCTTCTTTGTTCTGTTCTT -3’ |

**Figure**


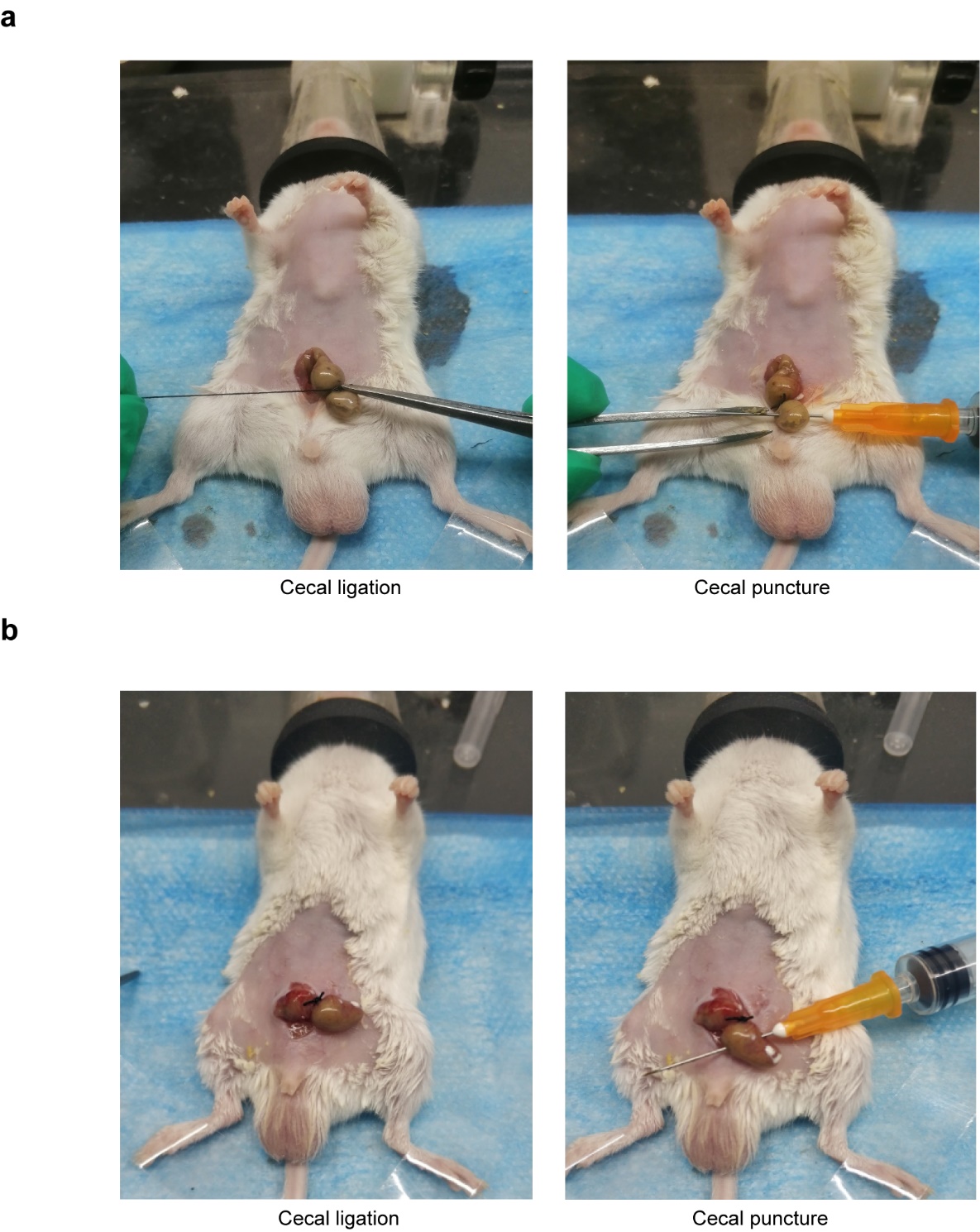


**Supplementary Figure S1.** Establishment of mouse CLP (a) and aggravated CLP models (b). The cecum was tightly ligated at 1/3 site from its end using 4-0 nylon suture, and double punctures of the cecal wall were performed with a 25 G needle. For the aggravated CLP model, the cecum was tightly ligated at 2/3 site from its end.


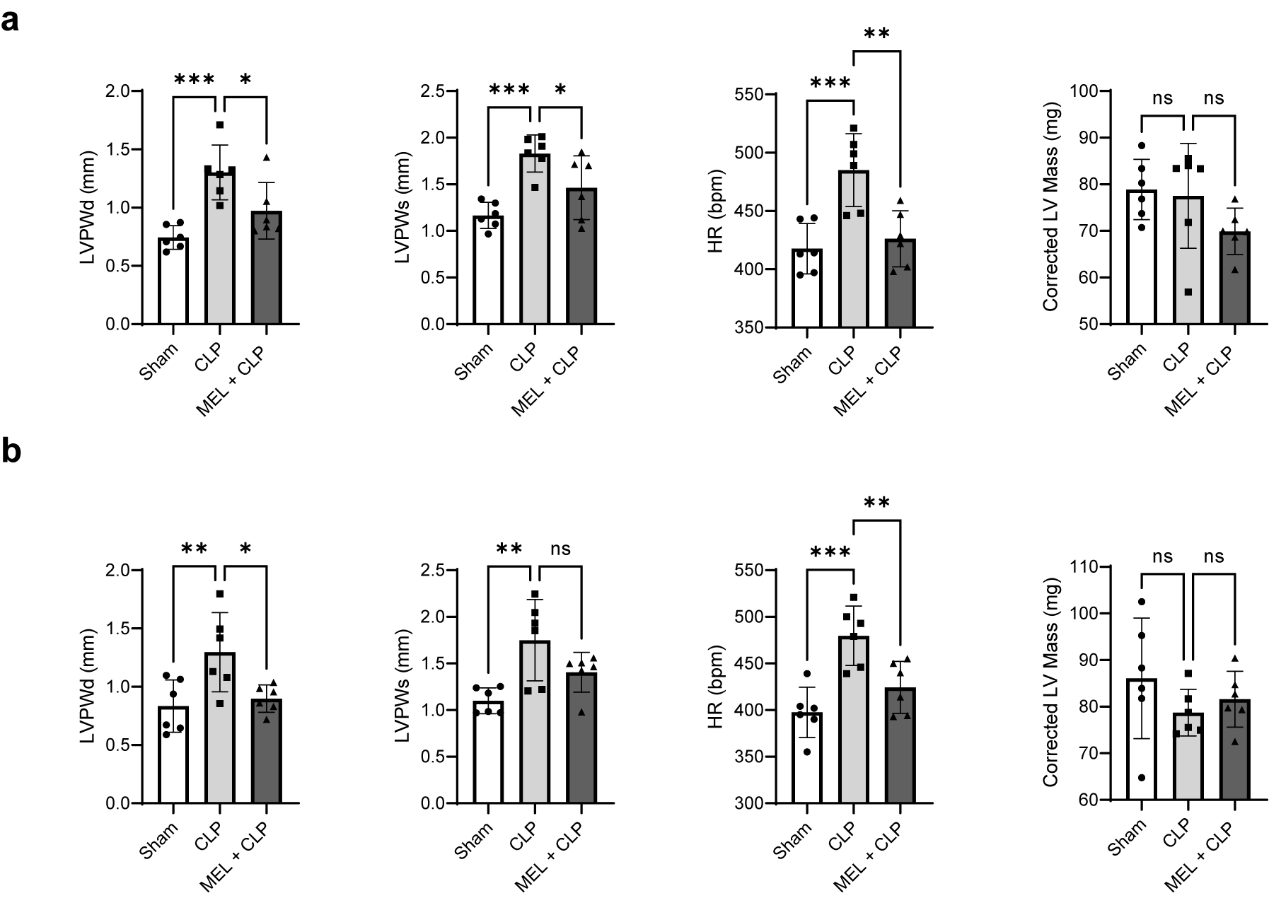


**Supplementary Figure S2.** Additional echocardiographic data of melatonin pretreatment in septic mice. (a) LVPWd, LVPWs, HR, and LV Mass statistical graphs of the long axis. (a) LVPWd, LVPWs, HR, and LV Mass statistical graphs of the short axis. ^*^*P*<0.05, ^**^*P*<0.01, ^***^*P*<0.001, ^****^*P*<0.0001 *vs.* Sham or *vs.* CLP; ns, non-signiﬁcant. n=6 for each group. Statistical analysis of data was performed using ANOVA.


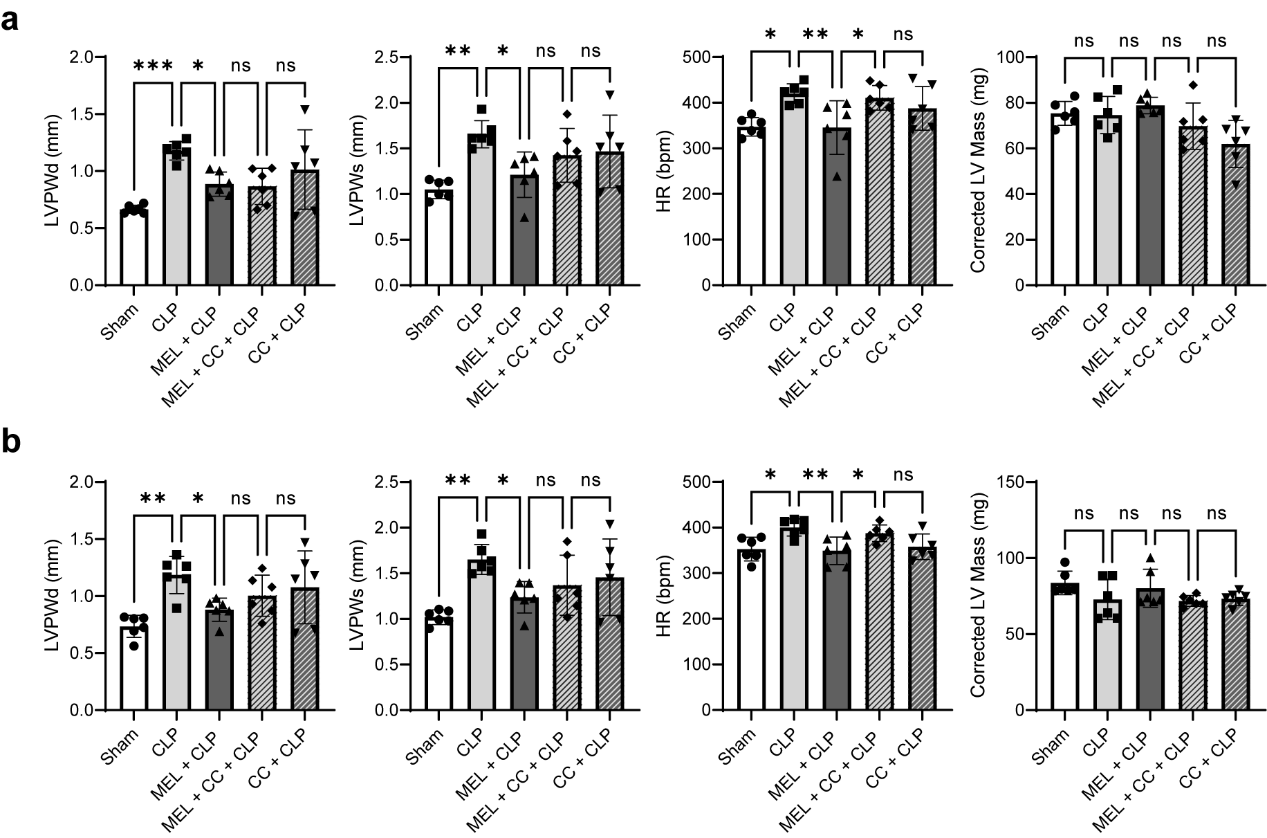


**Supplementary Figure S3.** Additional echocardiographic data on the effect of Compound C for melatonin pretreatment in septic mice. (a) LVPWd, LVPWs, HR, and LV Mass Statistical graphs of the long axis. (b) LVPWd, LVPWs, HR, and LV Mass statistical graphs of the short axis. ^*^*P*<0.05, ^**^*P*<0.01, ^***^*P*<0.001, ^****^*P*<0.0001 *vs.* Sham or *vs.* CLP or MEL + CLP; ns, non-signiﬁcant. n=6 for each group. Statistical analysis of data was performed using ANOVA.


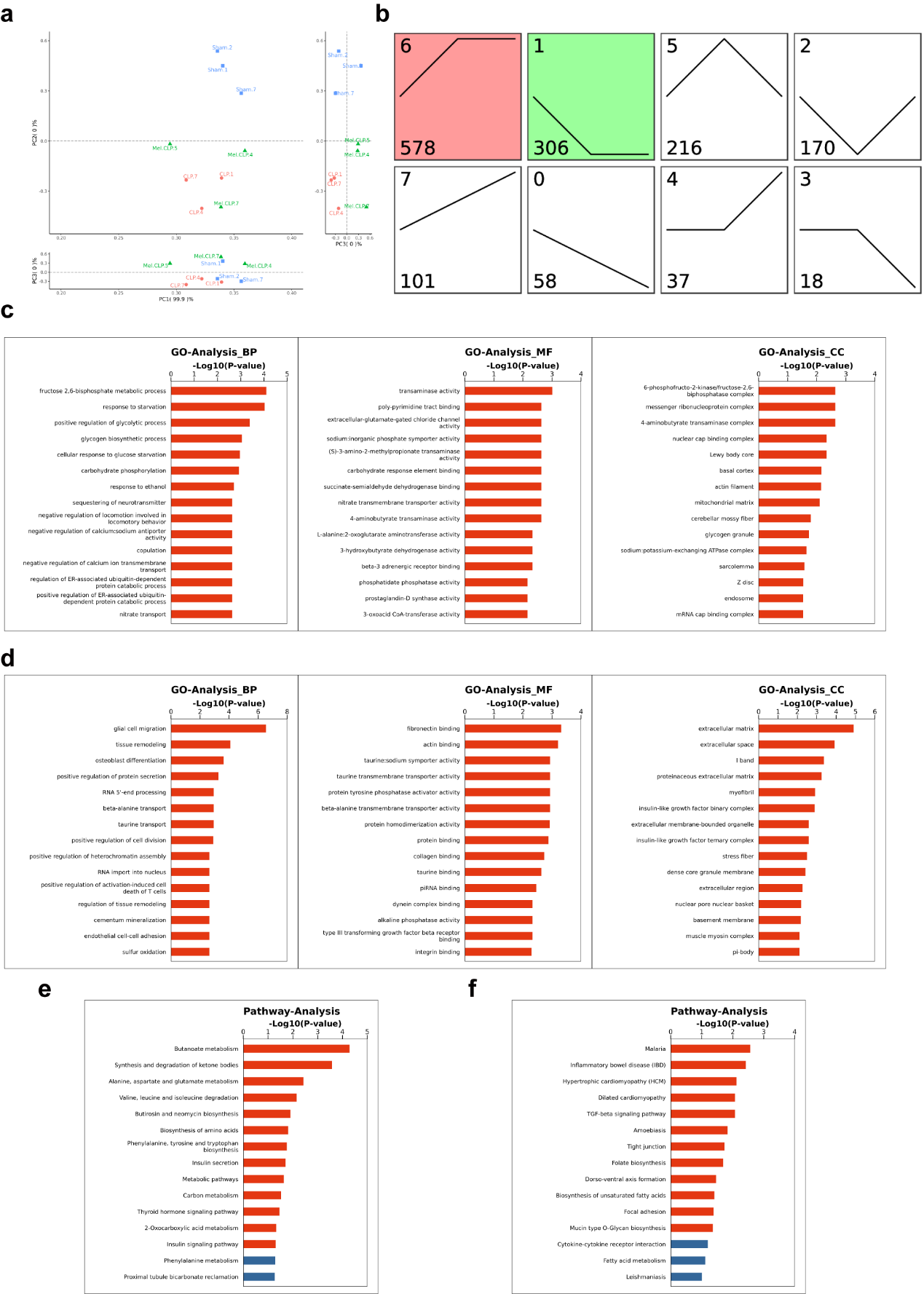


**Supplementary Figure S4.** Additional data of RNA-sequencing between the CLP group and the MEL+CLP group. (a) PCA diagram of each group. (b) Expression profiles in color indicated significant ones (*P*<0.05). Red indicated up-regulated and green indicated down-regulated. Profile number (up left), gene number (bottom left), and trend (line) in each profile were also labelled. (c) GO analysis of the up-regulated gene categories (*P*<0.05). (d) GO analysis of the down-regulated gene categories. (e) KEGG pathway analysis of up-regulated pathways (*P*<0.05). (f) KEGG pathway analysis of down-regulated pathways (*P*<0.05).


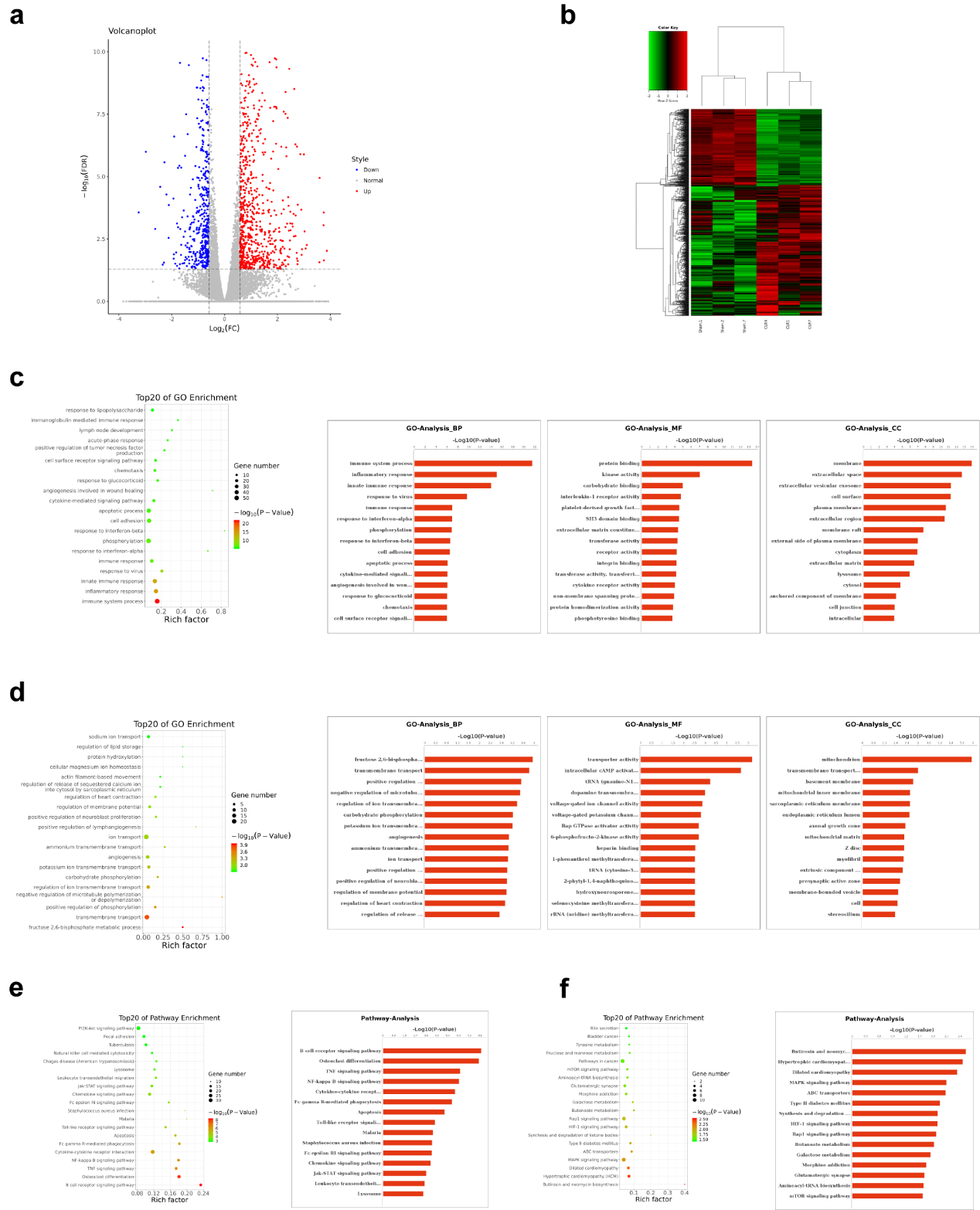


**Supplementary Figure S5.** Additional data of RNA-sequencing between the Sham group and the CLP group. (a) Differentially expressed mRNAs were displayed by volcano plot. The blue and red parts indicated >2 fold decreased and increased expression of the dysregulated mRNAs in cardiac tissues, respectively (*P*<0.05). (b) Cluster analysis of differential genes in the Sham group and the CLP group. (c) GO analysis of the up-regulated gene categories between the Sham group and the CLP group. (*P*<0.05). (d) GO analysis of the down-regulated gene categories between the Sham group and the CLP group. (*P*<0.05). (e) KEGG pathway analysis of up-regulated pathways between the Sham group and the CLP group. (*P*<0.05). (f) KEGG pathway analysis of down-regulated pathways between the Sham group and the CLP group. (*P*<0.05).


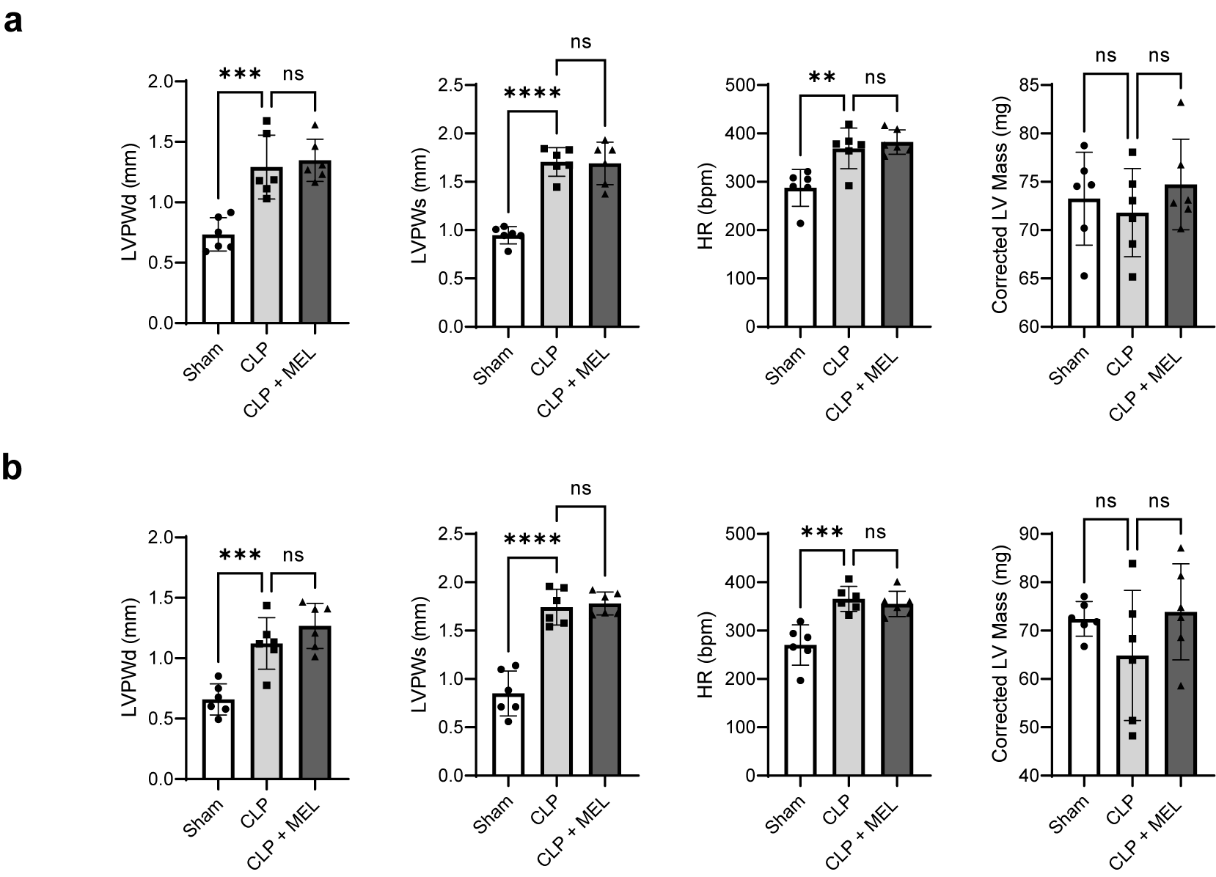


**Supplementary Figure S6.** Additional echocardiographic data of melatonin posttreatment in septic mice. (a) LVPWd, LVPWs, HR, and LV Mass statistical graphs of the long axis. (b) LVPWd, LVPWs, HR, and LV Mass statistical graphs of the short axis. ^*^*P*<0.05, ^**^*P*<0.01, ^***^*P*<0.001, ^****^*P*<0.0001 *vs.* Sham or *vs.* CLP; ns, non-signiﬁcant. n=6 for each group. Statistical analysis of data was performed using ANOVA.


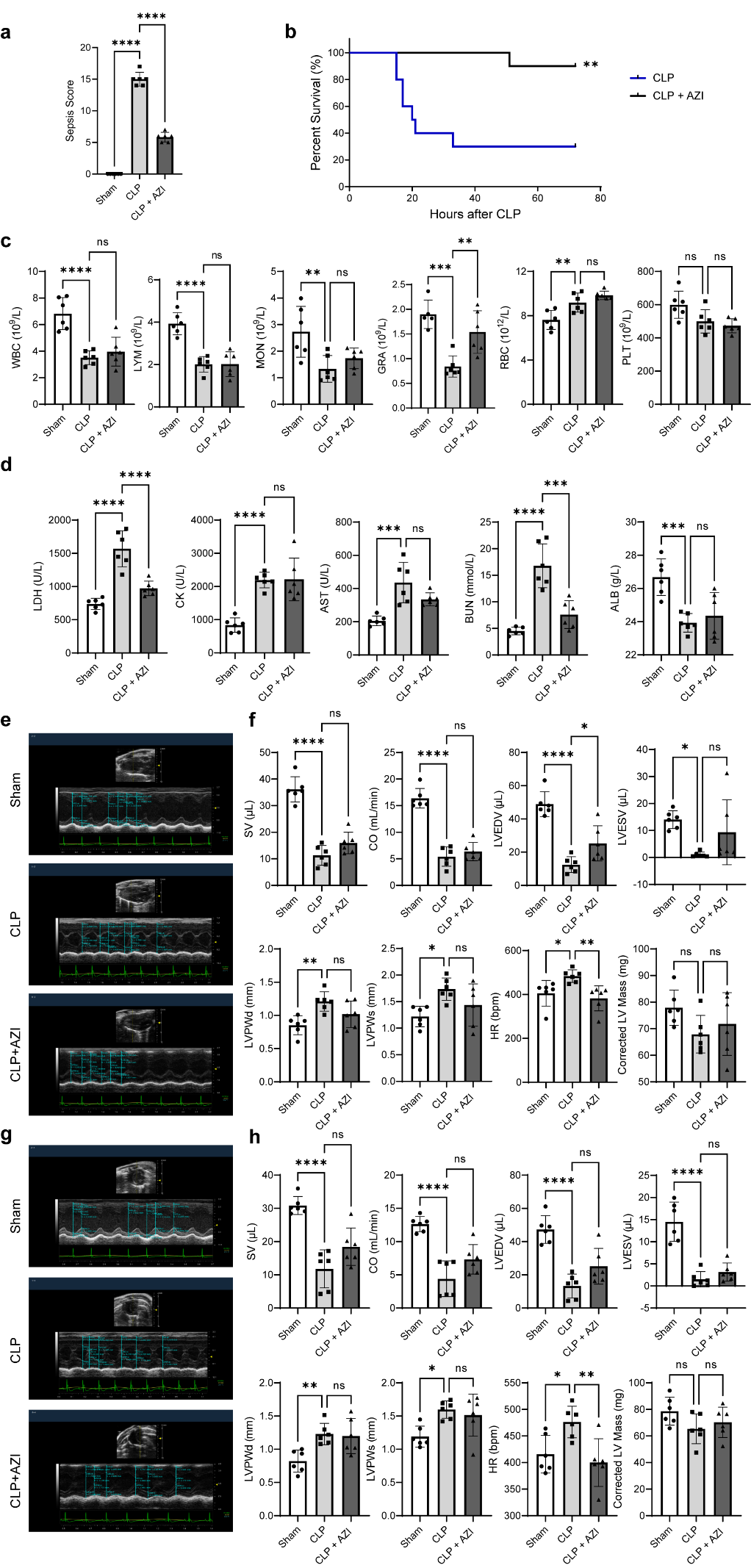


**Supplementary Figure S7.** Protective effect of azithromycin against CLP-induced myocardial injury in mice. (a) The sepsis scores. (b) KaplanMeier survival curves. Ten animals for each group were used for comparison. Mortality was observed within 72 h. (c) Blood routine parameters. (d) Blood biochemical parameters. (e) Representative echocardiography images of the long axis. (f) SV, CO, LVEDV, LVESV, LVPWd, LVPWs, HR, and LV Mass statistical graphs of the long axis. (g) Representative echocardiography images of the short axis. (h) SV, CO, LVEDV, LVESV, LVPWd, LVPWs, HR, and LV Mass statistical graphs of the short axis. ^*^*P*<0.05, ^**^*P*<0.01, ^***^*P*<0.001, ^****^*P*<0.0001 *vs.* Sham or *vs.* CLP; ns, non-signiﬁcant. n=6 for each group. Statistical analysis of data was performed using ANOVA.


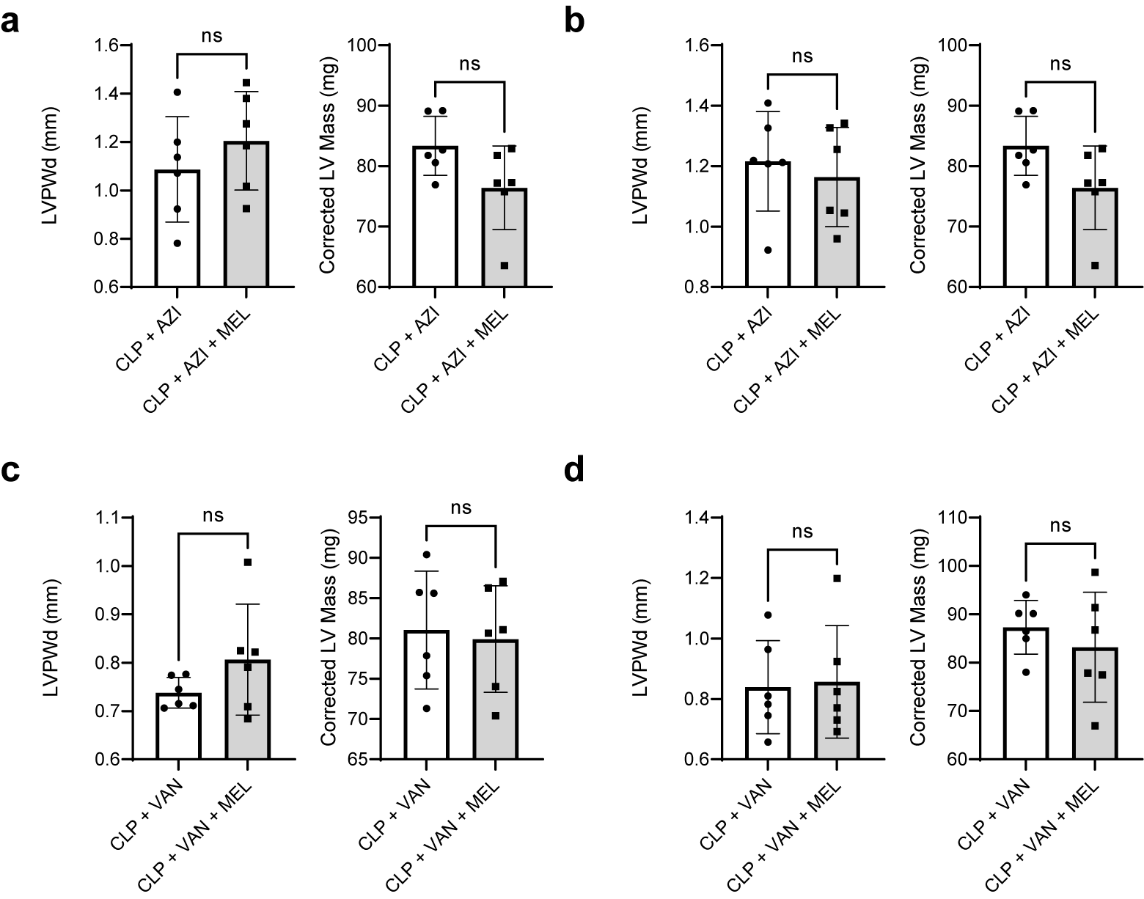


**Supplementary Figure S8.** Additional echocardiographic data of melatonin combined with azithromycin or vancomycin in septic mice. (a) LVPWd and LV Mass statistical graphs of the long axis. (b) LVPWd and LV Mass statistical graphs of the short axis. (c) LVPWd and LV Mass statistical graphs of the long axis. (d) LVPWd and LV Mass statistical graphs of the short axis. ^*^*P*<0.05, ^**^*P*<0.01, ^***^*P*<0.001, ^****^*P*<0.0001 *vs.* CLP + AZI or CLP + VAN; ns, non-signiﬁcant. n=6 for each group. Statistical analysis of data was performed using t-test.


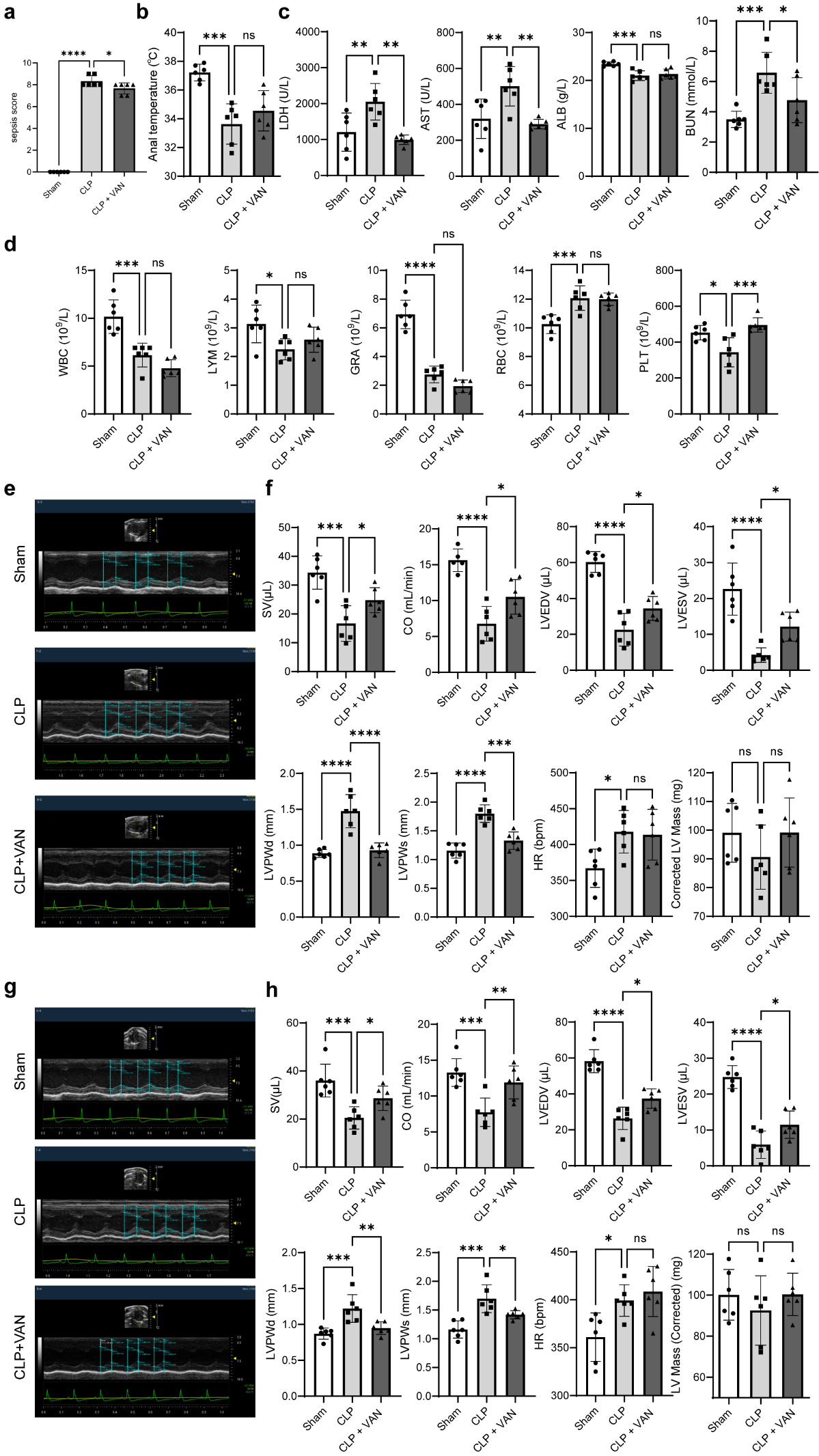


**Supplementary Figure S9.** Protective effect of vancomycin against CLP-induced myocardial injury in mice. (a) The sepsis scores. (b) The anal temperature. (c) Blood routine parameters. (d) Blood biochemical parameters. (e) Representative echocardiography images of the long axis. (f) SV, CO, LVEDV, LVESV, LVPWd, LVPWs, HR, and LV Mass statistical graphs of the long axis. (g) Representative echocardiography images of the short axis. (h) SV, CO, LVEDV, LVESV, LVPWd, LVPWs, HR, and LV Mass statistical graphs of the short axis. ^*^*P*<0.05, ^**^*P*<0.01, ^***^*P*<0.001, ^****^*P*<0.0001 *vs.* Sham or *vs.* CLP; ns, non-signiﬁcant. n=6 for each group. Statistical analysis of data was performed using ANOVA.

**References**

1. Kim D, Langmead B, Salzberg SL. HISAT: a fast spliced aligner with low memory requirements. Nat Methods. 2015;12: 357-360. <https://doi.org/10.1038/nmeth.3317>.

2. Anders S, Pyl PT, Huber W. HTSeq--a Python framework to work with high-throughput sequencing data. Bioinformatics. 2015;31: 166-169. <https://doi.org/10.1093/bioinformatics/btu638>.

3. Anders S, Huber W. Differential expression analysis for sequence count data. Genome Biol. 2010;11: R106. <https://doi.org/10.1186/gb-2010-11-10-r106>.

4. Benjamini Y, Drai D, Elmer G, Kafkafi N, Golani I. Controlling the false discovery rate in behavior genetics research. Behav Brain Res. 2001;125: 279-284. <https://doi.org/10.1016/s0166-4328(01)00297-2>.

5. Ashburner M, Ball CA, Blake JA, Botstein D, Butler H, Cherry JM, Davis AP, Dolinski K, Dwight SS, Eppig JT, Harris MA, Hill DP, Issel-Tarver L, Kasarskis A, Lewis S, Matese JC, Richardson JE, Ringwald M, Rubin GM, Sherlock G. Gene ontology: tool for the unification of biology. The Gene Ontology Consortium. Nat Genet. 2000;25: 25-29. <https://doi.org/10.1038/75556>.

6. Draghici S, Khatri P, Tarca AL, Amin K, Done A, Voichita C, Georgescu C, Romero R. A systems biology approach for pathway level analysis. Genome Res. 2007;17: 1537-1545. <https://doi.org/10.1101/gr.6202607>.
